# Untimely follow-up after cervical cancer screening: A qualitative study among healthcare providers in Flanders (Belgium)

**DOI:** 10.1371/journal.pone.0359860

**Published:** 2026-10-07

**Authors:** Marie Hendrickx, Laure-Anne Kerckhaert, Sara Willems, Rik Crutzen, Vera Verbestel, Katrien Vanthomme

**Affiliations:** 1 Department of Health Promotion, CAPHRI, Care and Public Health Research Institute, Maastricht University, Maastricht, The Netherlands; 2 Department of Public Health and Primary Care, Ghent University, Ghent, Belgium; 3 Department of Health Promotion, NUTRIM, Research Institute of Nutrition and Translational Research in Metabolism, Maastricht University, Maastricht, The Netherlands; Tarbiat Modares University Faculty of Medical Sciences, IRAN, ISLAMIC REPUBLIC OF

## Abstract

**Background:**

Cervical cancer remains a global health burden despite being largely preventable through vaccination, screening, and timely treatment. Delays in follow-up after abnormal screening undermine the effectiveness of organized cervical cancer screening programmes. In Flanders (Belgium), organized screening and reminder systems exist, yet 17.7% of women do not receive adequate follow-up after an abnormal Pap smear, highlighting the need to understand multilevel factors influencing timely follow-up.

**Methods:**

Between September and November 2025, 14 semi-structured group interviews were conducted in person with 120 general practitioners and gynaecologists responsible for cervical cancer screening in Flanders. Interviews were audio-recorded, transcribed verbatim, and analysed in MaxQDA, using iterative thematic analysis combining deductive multilevel framework coding with inductive insights. Interviews were double-coded, with discrepancies resolved through discussion. Data collection and analysis proceeded concurrently until saturation and findings were validated with stakeholders.

**Results:**

Despite the organized screening programme, healthcare providers experience cervical cancer screening follow-up as a complex, fragmented, and non-standardized multi-step pathway. Delays were mainly attributed to provider- and system-level factors, including inconsistent communication, challenges in interdisciplinary responsibilities and collaboration, high workload, and poorly integrated digital systems. Patient-related factors, such as language barriers and health literacy played a secondary role. Current follow-up processes were described as relying heavily on individual provider initiative rather than on well-coordinated, system-level structures. To improve timely follow-up, healthcare providers recommended standardised and proactive communication, clearer task allocation and responsibility, better integrated digital systems, the optimisation of the existing reminder mechanism and enhanced patient education.

**Conclusion:**

Delays in follow-up primarily reflect organisational and system-level weaknesses. Standardization, integrated digital systems, and clarified roles and responsibilities are key to reducing preventable delays. A “double safety net” combining provider oversight with informed patient involvement, alongside greater primary care engagement, may further enhance the continuity and timeliness of follow-up care.

## Introduction

Cervical cancer ranks fourth in incidence and cancer-related mortality among women worldwide [1]. In Belgium, it is the thirteenth most common female tumour in 2023 [2]. Social inequalities in cervical cancer incidence, morbidity and mortality are globally apparent and persist even within high-income countries [3]. This despite cervical cancer being preventable to a certain extent and curable when detected early [4].

In 2020, the World Health Organisation (WHO) launched the ‘*Global Strategy to Accelerate the Elimination of Cervical Cancer as a Public Health Problem’*, setting 2030 targets of 90% human papillomavirus (HPV) vaccination, 70% screening coverage, and 90% of women with precancerous lesions or cancer receiving effective treatment [5]. Achieving this treatment target depends, among others, on timely and appropriate follow-up after abnormal screening results and is therefore a critical component of cervical cancer elimination efforts.

In Flanders (a region in Belgium with 6.9 million inhabitants), an organized cervical cancer screening programme was introduced in 2013 for women aged 25–64 years, with a transition to primary HPV testing for women aged 30 and older since January 1^st^ 2025. For women aged 25–29, cytology screening every three years is still advised as primary screening method [6]. The screening programme uses a centralized invitation procedure and is organised by the Centre for Cancer Detection (CvKO). Women receive an initial invitation letter to consult their general practitioner (GP) or gynaecologist for a Pap smear, and those who do not respond are re-invited after 36 months. In 2024, 65.9% of the target population participated in screening (i.e., the latest data available until now) [7]. While gynaecologists still perform most Pap smears, the proportion conducted by GPs has steadily increased, reaching 14.5% in 2023 [7].

Screening programmes can directly reduce incidence and indirectly reduce disease-specific morbidity and mortality if every phase in the screening process is optimally performed: from informing and inviting the eligible population, to conducting the screening test, and ensuring timely and appropriate follow-up of an abnormal screening result [8]. Inadequate, referring to untimely and inappropriate, follow-up can undermine the benefits of screening, i.e., early detection, resulting in delayed diagnosis and poorer outcomes, but also increased psychosocial distress, and added costs to both patients and healthcare systems [9–11]. A meta-analysis estimated that 11.9% of the cervical cancer cases in countries with organised screening programmes are found among women with an inadequate follow-up after abnormal screening results [8]. In Flanders, the diagnostic follow-up rate for abnormal Pap smears remains below the WHO target of 90% so far. As both timeliness and appropriateness are essential dimensions of adequate follow-up, they represent conceptually different challenges within the screening pathway. In this study, we focus specifically on the timeliness of follow-up, as delays constitute a prerequisite barrier to effective and appropriate care.

To improve timeliness of follow-up, the Flemish government gradually implemented a fail-safe (reminder) mechanism from 2018 onward, beginning with the most severe cytological results [12]. This mechanism is applied to women aged 25–64 years registered with an abnormal screening result. If these women are not followed up within recommended time frames (i.e., 12 or 24 months depending on the severity of the cytological result), a letter is sent to the doctor who took the Pap smear (GP and/or gynaecologist). The mechanism’s performance improved between 2019 and 2020 with faster follow-up, and higher response to follow-up rates [13]. However, despite this positive evolution, 17.7% of women in Flanders still experienced delayed or absent follow-up in 2024 [7].

In-depth analyses from the Belgian Cancer Registry (BCR) and the CvKO demonstrate that follow-up rates in Flanders vary according to the cytological and associated HPV result [13]. In addition to clinical severity, international studies have reported associations between missing or delayed follow-up and lower socio-economic position [14–17]. Importantly, timely follow-up of abnormal cervical cancer screening results is not only determined by clinical severity and patient characteristics, but is also inherently linked to the actions of healthcare providers, clinical settings and the broader healthcare system. This is because the detection, communication and management of an abnormal finding rely on healthcare professionals’ engagement and the organisation of clinical care settings [18,19]. However, insight into factors influencing untimely follow-up originates mostly from low-income countries or the United States, while evidence from Belgium and the wider European context remains scarce [20].

Therefore, this study aims [1] to clarify how the follow-up pathway unfolds from the healthcare providers’ perspective, who plays a central role within the follow-up process as key intermediaries between patients, healthcare practices, and the wider healthcare system. In doing so, we furthermore aim [2] to identify factors that may contribute to untimely follow-up of abnormal cervical cancer screening results, and [3] to explore healthcare providers’ recommendations for improving timely follow-up care. To achieve these aims, this qualitative study adopts a multilevel analytical perspective that captures determinants operating at different, interrelated levels of the healthcare system [18].

Specifically, we distinguish four levels: 1) the patient level, capturing individual-level factors affecting follow-up behaviour; 2) the provider level, reflecting factors related to healthcare providers’ decision-making and communication practices; 3) the GP and gynaecology practice level, encompassing structural and organisational aspects, including practice size, location, resource availability, and staff capacity; and 4) the healthcare system and policy level, including the organisation of the cervical cancer screening programme, the fail-safe mechanism, and broader regulatory, financial and institutional contexts [18,21].

## Data and Methods

### Study design

This exploratory qualitative study is grounded in an interpretivist epistemological position, which holds that knowledge is inherently subjective and context-dependent. Rather than seeking a single objective account of the follow-up pathway, we aimed to understand how healthcare providers experience, interpret, and make meaning of this process within their specific professional and organisational contexts. To this end, semi-structured in-depth group interviews were conducted with healthcare providers responsible for cervical cancer screening in Flanders (Belgium) (i.e., GPs and gynaecologists). Group interviews were chosen as the primary data collection method for both methodological and practical reasons. Methodologically, the group format was considered particularly well-suited to exploring factors influencing follow-up, as peer discussion between clinicians can surface shared experiences, professional norms, and collegial dynamics that may not emerge as readily in individual interviews. Practically, interviews were organised through existing local quality assessment groups and professional associations, which are fixed, regularly meeting peer groups of healthcare providers, facilitating recruitment and enabling discussion within established professional relationships. One individual interview was additionally conducted with a GP working in a community health centre who expressed interest in participating but whose colleagues were not available to join. This interview was analysed together with the group data using the same analytical approach, as the topic guide and analytical framework were identical across all sessions. The themes identified in this interview were consistent with those raised in the group sessions, and no codes were unique to it, supporting its inclusion in the combined analysis. The data were analysed using a combined deductive-inductive thematic analysis approach. This approach allowed us to explore the pre-defined four-level perspective from the literature, while also capturing new insights from participants’ experiences. Accordingly, the themes generated through analysis reflect the researchers’ interpretation of participants’ perspectives, shaped by the analytical framework and the iterative dialogue between data and theory throughout the research process. This article was prepared in accordance with the consolidated criteria for reporting qualitative research (COREQ) [22]. The study was approved by the UGent/Ghent University Hospital Medical Ethics Committee (reference number: ONZ-2025–0232).

### Study population and recruitment

Eligible participants were GPs and gynaecologists actively involved in cervical cancer screening in Flanders. Recruitment was conducted through broad outreach targeting all GP associations across Flanders, and local quality assessment groups of GPs and gynaecologists, as well as the Flemish Organisation for Obstetrics and Gynaecology, ensuring geographic diversity across all provinces and natural variation in participant characteristics (gender, years of experience, practice setting). Participation was based on willingness and availability, introducing an element of convenience sampling. Purposive sampling was subsequently used to ensure inclusion of healthcare providers working in community health centres, as these settings are known to serve a disproportionately high share of vulnerable and socially disadvantaged populations and were not included yet in the sample. Participation was voluntary and based on written informed consent prior to the start of the interviews. Recruitment and data collection occurred iteratively, until no new factors were being identified during the interviews.

### Data collection

14 semi-structured interviews, of which 13 group interviews and 1 individual interview, were conducted in person, in Dutch (i.e., the main language in Flanders), with an average duration of 49 min, ranging from 31 to 68 min. No repeat interviews were conducted. In total, 120 healthcare providers participated, averaging 9 participants per group interview. Among these, one nurse (who assisted a participating GP), two retired healthcare providers and six healthcare providers in training participated, as they were still actively involved in the practice, networks or associations by contributing to cervical cancer screening activities. Participant characteristics are shown in Table 1, the nurse is included in the GP category, resulting in a combined total of 92 for that column. The sample achieved a broad geographical distribution across Flanders, with participants recruited from multiple provinces, and across care settings.

**Table 1:** Participant characteristics (n = 120).

	General practitioners and nurse (n = 92), of whom 91 GPs and 1 nurse in the GP practice	Gynaecologists (n = 28)
Gender		
Male	37	4
Female	55	24
Years of experience		
In training	4	2
≤5 years	16	11
6-10 years	30	1
11-20 years	19	3
≥21 years	23	11
Practice location		
Rural	62	6
Urban	30	22
Practice type		
Solo practice	19	2
Group practice	61	26
Community health centre	10	0
Other	2	0

All interviews were audio-recorded and took place between September 3 2025 and November 26 2025. Two researchers were present at each interview, with one facilitating the discussion (the main author, public health researcher, female, non-medical doctor, trained in qualitative interview techniques) and the other taking written field notes to document participants’ observations, including clues from non-verbal communication to capture data in natural circumstances. The study team who carried out the data collection had no professional or personal relationship with the participants. The research team's non-clinical identity shaped the interview dynamic in both directions: participants may have withheld information or simplified clinical detail assuming it was beyond the interviewer’s grasp, but several also spontaneously elaborated on or repeated clinical concepts in greater depth than they might have with a clinician interviewer, potentially enriching the data. Interviews were guided by a topic guide developed based on the study’s multilevel analytical framework, a review of the existing literature on follow-up of abnormal cervical cancer screening, and input from the stakeholder planning group. This group was recruited purposively through the professional network of the research team for the broader project, and comprised representatives from BCR, CvKO, an academic and a clinical GP, a gynaecologist, and two women who had previously received an abnormal cervical cancer screening result. Group members reviewed a draft version of the topic guide during a dedicated meeting, with opportunity to suggest additional topics and adapt existing questions. The revised topic guide was subsequently pilot tested by one GP from the group through a simulated interview, allowing further refinement before formal data collection commenced. The full topic guide is available in S1 Table. Participants were encouraged to reflect on experiences and perspectives across multiple levels of influence, including receiving abnormal results, communication of abnormal results to the patient, and their role and behaviour in follow-up care.

### Data analysis

Recordings were transcribed verbatim with the support of Scribewave, and subsequently checked and edited to ensure accuracy. Pseudonymization of the transcripts was performed manually by a researcher (MH). The transcripts were not returned to the participants for comments and/or correction.

Reflexivity was considered throughout the analytical process. The primary researcher entered the study with familiarity with the broader screening context and knowledge that 17.7% of women do not receive timely follow-up, though the specific patient-, provider-, practice-, and system-level reasons underlying these delays were largely unknown prior to data collection. An iterative thematic analysis was conducted in MaxQDA, based on the six-step systematic process described by Naeem et al. [23]. The multilevel framework (patient, provider, practice, healthcare system and policy) served as a deductive organisational structure, providing four pre-defined levels into which inductively generated codes were systematically assigned. Coding itself was conducted inductively, with themes and patterns emerging directly from the data and subsequently allocated to the most appropriate level of the framework. The inductive component in particular mitigated the influence of the researcher's preconceptions, as it allowed factors to emerge from the data itself rather than restricting the analysis to what was anticipated beforehand. Memos were used throughout the analytical process to document analytical thoughts, emerging interpretations, and code definitions, supporting transparency and consistency.

The coding frame was developed by two independent researchers (MH and LAK) of the study team based on approximately 10% of the data. The final coding frame, accompanied by code definitions, was then systematically applied to each interview. The coding frame with code definitions is available in S2 Table. Each interview was coded by the primary coder (MH) and double-coded by a second researcher (KV, VV or LAK). Inter-coder agreement was established through an in-depth line-by-line, code-by-code discussion of each transcript between the two coders. Segments where the coders had applied different codes were then discussed in detail and discrepancies were resolved through discussion to reach consensus, ensuring that interpretations remained grounded in participants’ own accounts rather than the researchers’ prior assumptions. Finally, broader themes were generated from the codes and mapped onto the four categories of the framework. Data collection and analysis proceeded concurrently. Data saturation was assessed inductively by tracking the generation of new codes across sessions. When no new codes were generated across two consecutive group interviews, it was determined that sufficient depth and breadth of information had been reached. All already planned interviews were conducted in full, but no additional interviews were scheduled once data saturation was reached. Findings were revalidated in a separate meeting with the stakeholder planning group, including policymakers and healthcare providers.

To enhance the trustworthiness of the findings, several strategies were applied following Lincoln and Guba [24] framework [25]. Credibility was supported through iterative concurrent data collection and analysis, double-coding of all transcripts with in-depth line-by-line consensus discussions, stakeholder revalidation of findings, and active researcher reflexivity throughout the process, in which the primary researcher’s prior knowledge of the screening context and non-clinical background were made explicit and revisited. Transferability was addressed through thick description of the study context and sample, and broad recruitment across all provinces of Flanders and across primary and secondary care. Dependability was strengthened through a systematic six-step thematic analysis process with a transparently developed coding frame and explicit code definitions, all available in S2 Table. Finally, confirmability was supported through the use of memos to document analytical decisions throughout the process, and stakeholder validation to verify that findings were grounded in participants’ accounts rather than researcher assumptions.

## Results

The results are organized according to the study’s three aims: describing healthcare providers’ experiences of the follow-up pathway from Pap smear collection to completion of follow-up, identifying multilevel factors contributing to delayed follow-up of abnormal results, and summarizing providers’ recommendations to improve timely follow-up care. Quotations from healthcare providers are translated into English and presented to support the summarised results.

### The follow-up pathway: from sample collection to completion of follow-up

Healthcare providers described follow-up after cervical cancer screening as a complex, multi-step pathway with multiple handovers and long time intervals. No standardised follow-up pathway was identified across interviews.

After collection by a GP or gynaecologist, Pap smears are sent to a laboratory, with results typically available within one to two weeks. Because of this delay, cervical screening results require prolonged monitoring, unlike, for example, most blood test results, which was repeatedly highlighted as a vulnerability in the pathway. Additionally, results often arrive in a fragmented manner, with HPV results preceding cytology, increasing the risk that results are overlooked or forgotten, potentially leading to delayed communication with the patient or untimely follow-up.

*“Sometimes we have that result after three days, but sometimes only after two weeks. But then, of course, it drops down? on the laboratory's website as the days go by, so we have to keep an eye on that.” (Female nurse)*

Multiple ways in which laboratory results are received were described by healthcare providers, depending on the laboratory and the administrative medical systems in use. They may appear on online platforms (e.g., LabOnline), requiring healthcare providers to actively consult the platform, or as a lab result or letter in different sections of the administrative system. Healthcare providers often mention that this lack of uniformity complicates result management and raises the risk of missed abnormalities. In addition, results are not routinely shared between all healthcare providers involved in the patient’s care. Results are normally communicated only to the requesting physician. The laboratory may also send the results to other healthcare providers, such as the patient’s GP, but only if this is explicitly indicated on the laboratory request form. Several providers indicated that this variability in inter-provider communication further increases complexity and uncertainty within the follow-up process.

*“And those results often end up in our documents, because it's an anatomopathological report. So I've had situations where I'm scrolling through the lab results and I can't find anything because it's in the document section.” (Male GP)*

Although patients can access results via Belgian e-health platforms (e.g., CoZo, *MijnGezondheid, MyNexusHealth*), healthcare providers consistently reported that most patients do not actively consult these platforms or are unaware that results are available there. As such, direct digital access to results was generally not perceived as a reliable mechanism for ensuring patient awareness of the result or timely follow-up. Furthermore, the medium through which healthcare providers communicated results to patients varied widely, most commonly by telephone, but also in person, by email, or by letter, depending on provider preference and practice organisation rather than standardized guidelines.

*“I always tell people to check the result in MyNexusHealth or CoZo [digital portals for sharing and accessing personal health records securely]. I always say that. Because I also feel that we should increase people’s responsibility. It's their health.” (Female gynaecologist)*

Furthermore, perceptions of who holds responsibility for organising follow-up care also varied. While some providers actively arranged immediate follow-up, such as colposcopy referral, longer-term follow-up (e.g., repeated Pap smear after 12 months) was often left to the patient, with considerable differences between providers.

*“But it's what you say, that deadline is harder to follow, even as a patient yourself. If you say the follow-up has to be done immediately, then you immediately take action. However, if duration to follow-up is longer, there is a greater chance of losing that patient, right? That's inherent to the Pap Smear […].” (Female nurse)*

Finally, awareness and knowledge of the existing fail-safe mechanism (i.e., reminders to healthcare providers on untimely follow-up) in Flanders was limited. Most healthcare providers were unaware of its existence, while those who were aware of it, had limited knowledge of its functioning. The fail-safe mechanism was rarely perceived as an active safeguard in daily practice.

### Factors influencing untimely follow-up of abnormal cervical cancer screening results from healthcare providers’ perspective

Across interviews, healthcare providers primarily attributed delayed follow-up to factors related to their own professional practices and to the organisation of the healthcare system. Patient- and practice-level factors were discussed less extensively and they were often framed as secondary influences rather than primary drivers. Findings therefore focus mainly on provider- and healthcare system and policy–level factors, while briefly outlining patient- and practice-level factors to reflect the multilevel nature of follow-up. The main themes identified at each level are presented in Fig 1.

**Fig 1:**
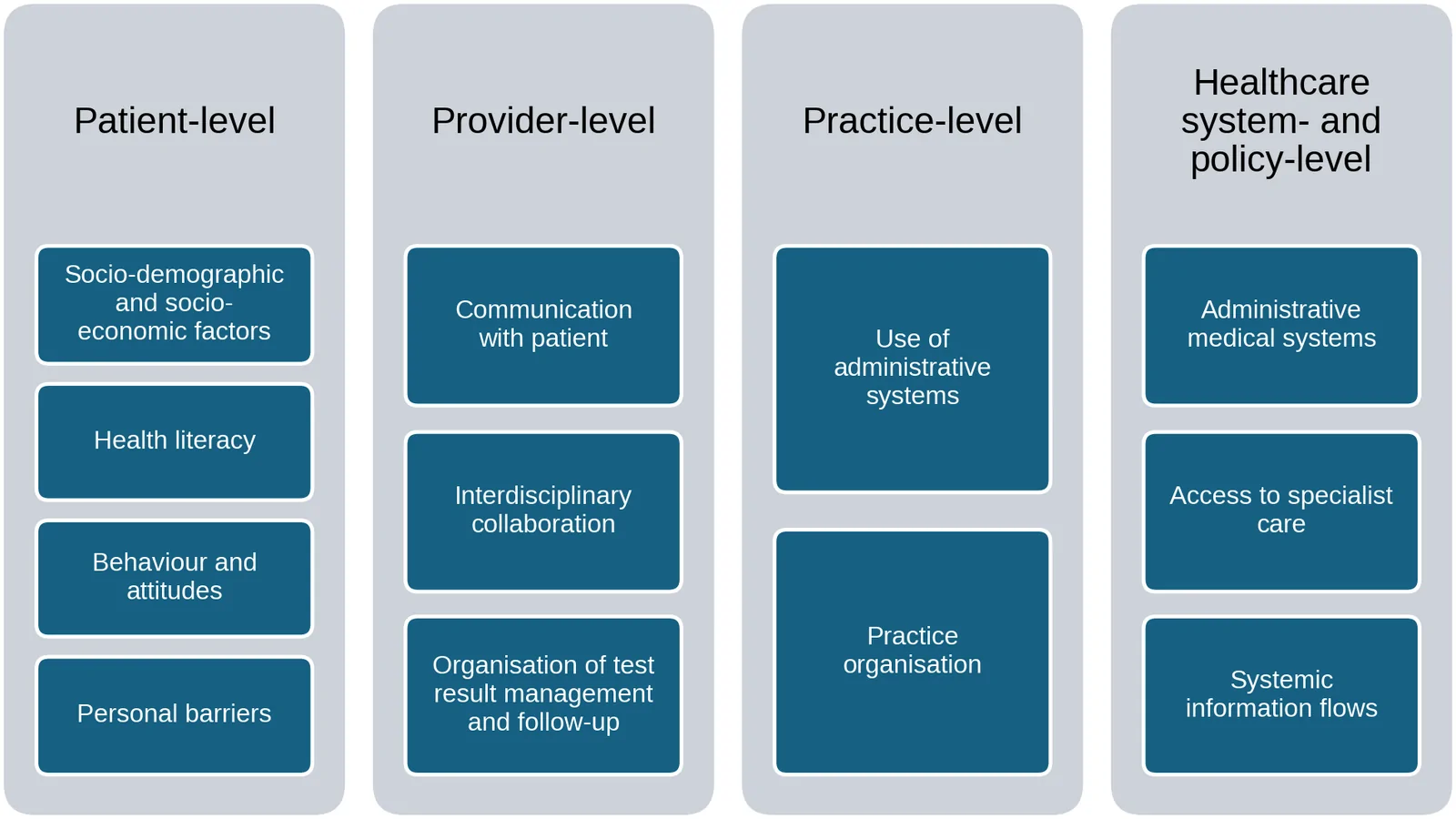
Main themes identified in interviews per level of the multilevel analytical framework.

### Patient-level factors

Regarding patient-related factors, language barriers and limited health literacy were most frequently mentioned, as they complicate communication of complex results and follow-up plans, particularly in a specialist care setting which is experienced as time-constrained. Healthcare providers had the feeling that when patients clearly know and understand their result and the necessity of follow-up, they are generally more likely to take appropriate action, highlighting the role of patient autonomy.

*“No, not consciously. I think that if a woman knows she has an abnormal Pap smear result, she will, and if she has been advised by her doctor to have it checked after six months or a year, she will do so. […] So I don't think there will be any patient factors. I think the main problem is that the message is not getting to the patient.” (Female GP)*

Conversely, limited understanding of abnormal results or required follow-up could hinder timely action, especially when responsibility for arranging follow-up is transferred to the patient. Fear for further (unpleasant) examinations and challenges in contacting patients (e.g., outdated contact details or repeatedly not replying to missed phone calls) were additional contributors to delay. Finally, providers noted that frequent GP consultations by patients reduce the risk of being lost to follow-up, by creating additional opportunities to detect and address follow-up in patients who might otherwise not be captured by existing follow-up mechanisms.

### Provider-level factors

A commonly reported practice, particularly among gynaecologists, was using a “no news is good news” approach. This practice was described as a way to manage workload by limiting the number of contacts with patients when results were perceived as reassuring or requiring no immediate action. However, this practice risks patients assuming results are normal when no contact is made, even if results are pending or have been overlooked, or in the case the healthcare provider failed to reach the patient (e.g., due to recently changed contact details). Providers acknowledged that the absence of proactive communication during the consultation in which the sample was collected, such as explaining possible results and how results will be communicated, can contribute directly to delayed follow-up.

*“I personally also let patients know about the normal Pap smears that I have performed myself, because I think it's a bit of a risky communication. Because if you haven't heard anything after a week, it's fine. But if you forget, the patient won't know anything either. And then I think it's risky to miss the rest.“ (Female GP)*

Healthcare providers also highlighted that cervical cancer screening follow-up is inherently complex and may hinder effective communication to patients, with multiple possible results and corresponding follow-up. It is often difficult to clearly explain to patients what an abnormal result means (i.e., that it is not (yet) cancer), why follow-up is needed, and the next steps.

Substantial variability existed in communication between gynaecologists and GPs. GPs were not systematically informed when a Pap smear was performed in specialist care, and vice versa, and communication of results and follow-up plans was inconsistent. Some routinely inform other healthcare providers involved, others communicate selectively or not at all. While gynaecologists sometimes avoided additional communication to limit GP workload, GPs expressed a clear preference for being informed to maintain oversight and continuity of care. Laboratory reports alone were considered insufficient, as they do not reflect clinical interpretation or intended management.

*“I think the biggest problem is that there is a lot of uncertainty about... for example, with breast cancer screening and colon cancer screening, with the iFOBT [i.e., immunochemical faeces occult blood test, colon cancer screening method], it is much clearer who initiates it and who follows up on it. And Pap smears are somewhat in a grey area. […] But I think it's mainly because it's not really clear, unlike mammography, which is a really clear programme. It's clear to doctors and it's clear to patients. And I don't think that's the case with Pap smears yet. It's not clearly defined.” (Female GP)*

Although healthcare providers agreed that the clinician who performs the Pap smear is responsible for communicating results to the patient, there was no consensus on whether this responsibility also includes actively ensuring that follow-up is arranged and completed. This ambiguity contributed to inconsistent practices.

*“I think especially when you receive the results yourself but you are not the one who took the Pap smear, I think that is very unpleasant. And then the question is, people go to the gynaecologist and you, as GP, receive the result, but you don't always know right away whether the gynaecologist has already communicated to the patient. Or was it supposed to be my responsibility to follow-up on that?” (Female GP)*

Healthcare providers described relying heavily on individual organisation strategies, such as agenda notes, leaving documents open as prompts, or reminders in electronic patient records, to manage pending results and follow-up. While helpful, these systems were informal and dependent on personal vigilance. Furthermore, documentation was often motivated by medico-legal considerations (e.g., ensuring traceability and liability coverage) rather than being designed to actively support structured and systematic follow-up.

A high and increasing workload further heightened vulnerability. Providers described reviewing large volumes of laboratory reports and making repeated contact attempts as time-consuming and cognitively demanding. Under such conditions, abnormal results may be overlooked.

*“If they are abnormal. Yes, I read those results and I always adjust my schedule accordingly. Because if you ever forget to remind people that it's time for another smear test, so when they come in and you see that it's almost time in your schedule, then I'll tell them [...].” (Female GP)*

### Practice-level factors

Practice-level factors included differences in inbox structures, system integration and filtering options that complicated result tracking, particularly in group practices with shared mailboxes. Limited possibilities to schedule long-term follow-up appointments in advance often automatically shifted responsibility to patients.

*“Only those with abnormal cytology results who need to have a colposcopy immediately are given an appointment straight away. Those who need an appointment in one year don't get an appointment with me, they know... But then again, they have a telephone number, but I don't know...” (Male gynaecologist)*

Furthermore, few GP practices had dedicated other staff, such as a specialized nurse or administrative personnel, to perform Pap smears, manage screening results and coordinate follow-up appointments. Healthcare providers attributed this primarily to financial constraints, and several participants noted that having such dedicated support would, in an ideal situation, improve the reliability and continuity of follow-up care.

### Healthcare system and policy-level factors

As noted above, healthcare providers reported that delayed and fragmented result reporting, along with limited standardisation in how digital messages were entered into medical record systems, complicated retrieval and interpretation. In addition, follow-up reminders were typically only visible when actively opening a patient record, limiting their effectiveness when patients did not return regularly for another consultation, which was not guaranteed, particularly in a relatively young population.

Although digitalisation has replaced paper-based communication, providers felt it often increased fragmentation. Information is scattered and must be tracked across multiple e-health platforms and systems (e.g., laboratory portals, hospital and/or primary care medical record systems, secure health information exchange platforms such as CoZo and secure messaging systems used for communication between health care providers such as the eHealth box), requiring additional administrative effort. Important notifications, such as letters of the fail-safe mechanism, may be missed when sent via rarely consulted platforms (e.g., eHealth box).

*“But come on, I mean, now that responsibility is being made so difficult for us, because you get results from your own laboratory, you get results from hospitals X, Y and Z, all in different ways. Yes, that's not okay.” (Male GP)*

Long waiting times for gynaecology consultations, sometimes up to several weeks, were perceived as structural barriers. Delays in securing colposcopy appointments could reduce patient motivation and create uncertainty regarding responsibility once referral had been made but timely access was not guaranteed.

*“You can let your patient know that there is a problem, but you have to make a gynaecological appointment, we do not perform this follow-up care. But then it's usually up to the gynaecologists, isn't it? How long do they have to wait before they can see a gynaecologist? Yes, and that's where I think things often go wrong. And the question is, who is responsible for that?” (Male GP)*

Digital health platforms present mixed challenges as part of the follow-up process. While many patients do not consult their results, limiting the platforms’ effectiveness, others do access their results but often without contextual explanation, which can lead to confusion, anxiety, and additional provider workload.

Laboratory-generated follow-up recommendations represent an additional source of tension. GPs tend to rely on these recommendations, whereas gynaecologists often considered them potentially inaccurate because they do not always account for the patient’s full screening history. When such recommendations were also visible to patients alongside their results via digital health platforms, discrepancies with specialist advice could create confusion and contribute to delayed or inconsistent follow-up.

*“I'm not really sure how laboratories work, because it's done via an automated system, that they write a conclusion. Because, of course, it depends on your HPV type and then it also depends on whether or not cytology is performed. But I'm not really sure how they do that. But it's certainly not based on the patient per se, on their medical history. So it would definitely be better if they didn't give any advice.” (Female gynaecologist)*

### Healthcare providers’ recommendations to improve timely follow-up

Finally, throughout the interviews healthcare providers formulated recommendations to improve timely follow-up across four themes: 1) proactive and standardized communication to patients, 2) clear task allocation and responsibility between healthcare providers, 3) integrated and transparent digital systems for patients and healthcare providers, and 4) optimising the fail-safe mechanism.

### Proactive and standardized communication to patients

Healthcare providers emphasized the need to better prepare patients at the time of Pap smear collection by clearly explaining possible results and follow-up pathways. Standardising this core message across professionals was seen as a way to reduce variability and misunderstanding. Providers also underscored the importance of strengthening patient knowledge about cervical cancer screening processes and follow-up requirements, so that patients are better equipped to act on results when received. In addition, participants supported automatic digital notification of results via secure platforms, provided that the messages do not contain raw laboratory results, but rather a clear, comprehensible message.

*“I think what could also be interesting is if the laboratory sends the results to the patient and includes some information in understandable language explaining what LSIL means, what HSIL means, what ASCUS means... but in plain language.” (Female GP)*

Standardized notifications advising patients on next steps (e.g., contacting the Pap smear-taker in case of abnormal results) were viewed as a strategy to enhance knowledge and reduce delays.

### Clear task allocation and responsibility between healthcare providers

Participants highlighted persistent ambiguity regarding roles and responsibilities across primary and secondary care. It was often mentioned, mostly by GPs, that the initial Pap smear could preferably be performed in primary care, with gynaecologists taking a more prominent role in follow-up when abnormalities were detected.

*“But then again, regarding Pap smears, I believe gynaecologists have responsibility that go beyond prevention. And I think preventive screening is primarily something that can be managed by the GP.” (Female GP)*

While most agreed that the clinician performing the Pap smear should communicate the result, responsibility for ensuring follow-up was less clearly defined. Providers advocated for explicit agreements and more systematic, ideally automatic, information exchange between GPs and gynaecologists, with several emphasising a coordinating role for the GP in maintaining continuity and oversight.

### Integrated and transparent digital systems for patients and healthcare providers

Improving digital infrastructure was considered essential. Suggested measures included automated patient reminder systems at predefined intervals depending on the type of the abnormal result (e.g., 6, 9, 12, or 18 months) and the integration of a structured overview module within electronic medical records summarising screening history, results, and follow-up status for each population-based screening programme. This centralized overview would allow providers to quickly assess outstanding actions and reduce reliance on individual memory or ad hoc tracking. Some participants also mentioned automated alerts or artificial intelligence–based “pop-up” notifications that flag delayed follow-up. These tools were seen as a means to support clinical vigilance and to reduce the risk of cases being overlooked.

*“Ideally, an abnormal Pap smear should be coded in such a way that it is automatically entered into our system and incorporated into our programme and schedule. […] In other words, it should happen automatically.” (Female GP)*

In addition, several providers pointed to low patient familiarity with digital health platforms as a practical barrier, and suggested that encouraging patients to actively consult these platforms, for instance to access their own results, could serve as an additional safeguard against missed follow-up and foster a greater sense of shared responsibility in the follow-up process.

*“Can't the patient also read the results in MyNexusHealth [i.e., digital portal for sharing and accessing personal health data securely] or something similar? […] Couldn't we encourage them to check it and contact their GP if they have any questions?” (Female GP)*

### Optimising the fail-safe mechanism

Finally, although familiarity with the existing fail-safe mechanism was limited among most healthcare providers, its potential was broadly valued, and notably, several healthcare providers independently suggested the need for a similar mechanism without being aware that one already existed. The fail-safe system was rarely perceived as an active safeguard in daily practice, and providers generally agreed that improvements would be needed before it could fulfil this role. Participants recommended extending fail-safe notifications to patients as well as providers, which was seen not only as a way to reduce the risk of delayed or absent follow-up by creating a “double safety net”, but also as a means to encourage shared responsibility between patients and healthcare providers in the follow-up process.

*“But I think that that fail-safe mechanism will be useful once it is finalised. But then, yes, it would be good if it also ends up in the patient's e-box, for example, [...].” (Female gynaecologist)*

## Discussion

This study investigated how the follow-up pathway for abnormal cervical cancer screening results is experienced by healthcare providers, which factors contribute to untimely follow-up according to healthcare providers, and how timely care can be improved. Overall, follow-up after abnormal cervical cancer screening is not perceived as a standardised pathway, but a fragmented, multi-step process with multiple handovers and prolonged intervals. According to healthcare providers, delayed follow-up is primarily driven by provider- and healthcare system and policy-level factors, rather than patient-level factors. This limited emphasis should be treated with caution as it reflects providers’ views only, may be subject to social desirability bias and self-serving attribution, as further discussed in limitations, and may therefore not fully capture patients’ perspectives. Additionally, findings from international studies indicate that socioeconomically disadvantaged women often experience longer delays in diagnostic follow-up and treatment after abnormal screening results [9,14,26–28]. However, in general, Belgian evidence on patient-level determinants of follow-up remains non-existent. Language barriers were one of the few patient-related factors raised by healthcare providers, which aligns with previous research indicating that language concordance between patients and providers can improve the timeliness of follow-up, particularly among disadvantaged populations [29].

Although patient-level factors were less prominently emphasized, limited familiarity with abnormal screening results and follow-up procedures emerged as a relevant underlying barrier. In this context, participants highlighted the importance of strengthening patient knowledge of the cervical cancer screening programme and their specific results and follow-up requirements, through proactive communication at the time of Pap smear collection, so that patients are better equipped to act on results when received. This is also supported by previous research showing that targeted educational interventions can improve adherence to follow-up recommendations [30–32]. This does not imply that health education needs to be promoted as a standalone solution to improve timely follow-up [33,34]. However, participants argued that in the context of the inherent complexity of the cervical cancer screening programme, compared to more straightforward diagnostic pathways in other cancer screening programs (e.g., colon and breast cancer screening), strengthening patient knowledge is particularly relevant. While encouraging patients to take an active role in their care may improve engagement, this also raises a tension between promoting patient responsibility and maintaining clear accountability within the healthcare system. Several participants therefore advocated a “double safety net” approach, combining provider oversight with informed patient involvement, including through the use of digital health platforms, as a more balanced model of shared responsibility [35]. Within this perspective, participants suggested that fail-safe notifications or reminders should not only be directed to healthcare providers but also be communicated to patients, enabling them to actively participate in ensuring follow-up. This model recognizes that while structural safeguards are essential, enhancing patient understanding can provide an additional, complementary layer of protection against delayed or missed follow-up [32].

Healthcare providers consistently pointed to fragmented communication across all levels, variability in how results are transmitted and documented, and uncertainty regarding who is responsible for ensuring that follow-up is completed. This ambiguity is also reflected internationally. For example, WHO guidance generally refers to providers responsible for follow-up, without specifying whether this refers to a GP, gynaecologist, nurse or other healthcare professional [36]. In practice, the organisation of cervical cancer screening still varies considerably across European countries [37]. For example, in some settings such as the Netherlands, Denmark, and the United Kingdom, initial Pap smears are primarily performed in primary care, whereas in countries such as Germany, France and Belgium they are more commonly carried out by gynaecologists [37,38]. Currently, in Flanders, 86% of cervical smears are performed by gynaecologists [7]. Within this context of fragmented responsibilities, participants also cautioned that when follow-up processes are not clearly assigned within the healthcare system, responsibility may implicitly shift to patients, which can exacerbate existing patient-level barriers and increase the risk of delayed follow-up. Against this background, the suggestion of participants to shift initial screening more systematically to primary care deserves consideration. GPs are in regular contact with a broad cross-section of the population, placing them in a favourable position to offer cervical cancer screening. To address concerns regarding GP workload and sustainability, screening could also be delegated to adequately trained primary care professionals (e.g., nurses, midwives, or practice assistants) [39,40]. In addition, self-sampling strategies may further complement this approach by increasing accessibility and participation, particularly among under-screened populations, while requiring careful organisation of follow-up pathways to ensure timely management of positive results [41–44]. Greater involvement of primary care staff in initial screening, an approach already implemented in several European countries, may therefore alleviate pressure on specialist services, potentially reducing waiting times and costs, while also contributing to increased screening coverage and reduced inequalities [45,46]. In such a model, gynaecologists would retain a central role in the management of abnormal results and more complex cases following referral. This clearer delineation of responsibilities between primary and secondary care could help address the coordination challenges identified in this study and support more timely and consistent follow-up pathways [47].

Finally, the interviews were conducted during the transition to primary HPV screening introduced in Belgium on January 1^st^, 2025 [6]. During this period, healthcare providers were still becoming familiar with the new screening approach, and many reflections therefore referred to experiences with the previous screening system. However, the issues raised largely relate to the broader organisational and communication processes within the follow-up pathway, than to the screening test itself. Several of the recommendations mentioned by participants have been reflected in ongoing policy developments, such as more streamlined laboratory communication using internationally standardized messaging formats for electronic health information (e.g., Fast Healthcare Interoperability Resources developed by Health Level Seven; HL7‑FHIR), automatic result transmission to GPs once complete laboratory results are available and plans for automatic communication of screening results to women [48]. These developments are also consistent with evidence from intervention studies demonstrating that more centralized follow-up systems and standardized reminders can improve follow-up after abnormal screening results [49]. Importantly, timely follow-up remains an essential component of screening under primary HPV screening as well. This is particularly relevant given that initial rounds of HPV screening may be associated with higher referral rates, highlighting the importance of adequately identifying and addressing potential barriers to follow-up [50].

### Strengths and limitations

While this study focused specifically on healthcare providers’ perspectives and did not include patient experiences, we recognize the important value of patients’ viewpoints. However, focusing on providers allowed for detailed insight into clinical workflows and professional decision-making, which can later inform patient-focused research. Although most cervical smears are performed by gynaecologists in Flanders [7], the sample included more GPs than gynaecologists, partly due to challenges in recruiting specialists. To mitigate this risk of disproportionately shaping the findings, the topic guide was applied flexibly during interviews with gynaecologists, with the facilitator actively probing topics and perspectives that had emerged prominently in GP interviews but had not yet been spontaneously raised, ensuring that all key themes were meaningfully explored across both provider groups. Although broad outreach across all GP associations and gynaecological networks in Flanders ensured wide geographic coverage and natural variation in participant characteristics, recruitment was primarily based on willingness and availability. Providers more motivated or engaged with the topic may have been more likely to participate, potentially introducing self-selection bias. This should be considered when assessing the transferability of the findings. Nevertheless, including a large number of participants across both primary and secondary care ensured diverse viewpoints and enhanced the robustness of the findings. Although the group interview format was chosen deliberately to stimulate peer discussion and capture shared professional perspectives, it may also have introduced group dynamic effects. Social desirability within professional peer groups may have led some participants to align their responses with dominant views, avoid disclosing practices deviating from professional norms, or attribute delays to external system- and policy-level factors rather than to their own clinical practice. Anchoring effects, whereby early contributions from more vocal or senior participants shape the direction of subsequent responses, cannot be excluded. While the presence of a second researcher taking field notes allowed for some observation of non-verbal communication and group dynamics, these potential influences should also be considered when interpreting the relative emphasis placed on different levels of the multilevel framework. The findings are context-specific and embedded within the organisational and digital infrastructure of the Flemish healthcare system, which may limit transferability to other settings. However, by applying a broad recruitment strategy across levels of care and across all provinces of Flanders, the study captures a wide range of perspectives, enabling the identification of system-level vulnerabilities and coordination challenges. For example, inconsistencies in result reporting between hospital-based and private laboratories or follow-up recommendations that diverge from official national guidelines (as issued by Sciensano in Belgium) within specific practices or professional associations. Finally, as a qualitative study, the results are not intended to quantify the frequency or magnitude of factors influencing untimely follow-up, but still offer critical insights to inform improvements in real-world clinical practice.

### Future research

While this study provides insight into organisational and professional determinants of delayed follow-up, understanding how women experience communication, responsibility, and navigation of follow-up care after an abnormal cervical cancer screening result is essential. Therefore, future research should focus on incorporating patient perspectives. In addition, quantitative research in the Belgian and broader European context is needed to complement these qualitative insights by identifying patient-, provider-, and system-level factors associated with delays. Particular attention should be given to potential social inequalities in follow-up care, which were not prominently raised in this study but may influence access to and timeliness of follow-up [9]. Finally, potential approaches to improve timely follow-up, such as standardized communication protocols, integrated digital reminders, or dual reminder (fail-safe) notification mechanisms, could be further explored with patients, health care professionals and policy makers and, where appropriate, implemented and evaluated for effectiveness. Insights from such studies may help to inform their possible adaptation and transferability to other healthcare settings and regions, thereby contributing to efforts to enhance timely follow-up and the reliability of cervical cancer screening programmes.

## Conclusions

According to healthcare providers, the follow-up pathway after abnormal cervical cancer screening results is experienced as a complex, fragmented, and non-standardised multi-step process, characterised by multiple handovers, prolonged intervals, and heavy reliance on individual provider initiative rather than coordinated system-level structures. Untimely follow-up is primarily a consequence of fragmented communication, unclear task allocation and responsibilities, and insufficiently integrated digital systems. Strengthening coordination, standardizing communication, clarifying accountability, and further improving the existing fail-safe mechanism within routine workflows are essential steps toward more reliable follow-up. A “double safety net” combining provider oversight with informed patient involvement, supported by proactive communication and the use of digital health platforms, may further enhance continuity and timeliness. Greater engagement of primary care in initial screening may additionally contribute to reducing coordination challenges and preventable delays. These findings remain relevant in the context of the ongoing transition to primary HPV screening, where timely follow-up continues to be essential.

## Supporting information

S1 Table Topic guide interviews with healthcare providers. (PDF)

S2 Table Coding frame with code definitions – interviews with healthcare providers. (PDF)
