# Supplementary material for: Untimely follow-up after cervical cancer screening: A qualitative study among healthcare providers in Flanders (Belgium)
Source: PLoS One. 2026 Oct 7;21(10):e0359860. doi: 10.1371/journal.pone.0359860 (PMC13645050; doi:10.1371/journal.pone.0359860)
Supplement: Supplementary file 1 [file pone.0359860.s001.pdf]

S1. Table. Topic guide interviews with healthcare providers.

[Total duration: 50-90min, depending on type (individual interview or interview with multiple interviewees)]

Introduction of the interview and researcher

Objectives: To introduce the interview and researcher, make the participant(s) feel comfortable, explain the study's purpose and ensure informed consent. ***[individual: 5min; multiple: 5-10min]***

- Welcome the participant(s) and introduce yourself (informal part of the interview)
- Thank the participant(s) for participating in the interview and study
- Introduce the purpose and course of the interview, make sure there is room for questions if needed
- Let the participant(s) sign the written informed consent form (if not already done so)
- Ask permission to start recording & start recording

Background information of the participant(s)

Objective: To gather contextual information on the healthcare provider's background, experiences and role in cervical cancer screening and follow-up. ***[individual: 5min; multiple: 10min]***

**Topics**	**Examples of questions**	**Probes**
Professional background and experience	- Can you describe your professional background?<br>- What proportion of your work involves cervical cancer screening and follow-up care?<br>-	- Sex<br>- Role, years of experience<br>- Have you worked in different types of healthcare settings?
Practice setting	- Can you describe your practice setting?<br>- How often do you encounter patients with abnormal cervical cancer screening results?	- Type of practice, practice setting (e.g., interdisciplinary work environment, private practice, (university) hospital, urban/rural, patient demographics)

General open questions to start conversation

1. Please walk me through the usual process of (abnormal) cervical cancer screening results?
2. Currently, around 1 in 5 women with abnormal cervical cancer screening results in Flanders do not receive timely follow-up – either it is delayed or it does not happen at all. In your experience, what do you think are the main factors contributing to this untimely follow-up? ***[individual: 10min; multiple: 15min]***

Communication of abnormal CCS results [Patient and provider level]

Objective: To explore healthcare providers' experiences with and perspectives on receiving and communicating abnormal cervical cancer screening results. ***[individual: 5min, multiple: 10min]***

**Topics**	**Examples of questions**	**Probes**
Standard communication practices	- How are abnormal cervical cancer screening results communicated towards patients in general?	- How do you receive results?<br>- Do you provide results in-person, by phone or electronically?<br>- If electronically, who takes the initiative (e.g., difference between sending email or result automatically sent to patient through eHealth platform)?
Challenges in delivering results	- What challenges, if any, do you encounter when informing patients about abnormal results?	- Are there personal (e.g, emotional or psychological) factors that impact these conversations?<br>- Are there specific patient groups for whom communication is difficult? How do you handle this?
Adaptations of communication strategies	- How do you tailor your communication approach based on individual patient characteristics?	- Do you modify your approach based on patient characteristics (e.g., health literacy level)?

Informing and guiding follow-up of abnormal CCS results [Patient, Provider and Practice level]

Objective: To identify healthcare providers' experiences with and perspectives on (un)timely follow-up care and the potential influence of individual patient and provider, as well as practice organization characteristics on timely follow-up of abnormal cervical screening results.
[individual: 10-15min; multiple: 15-20min]

Topics	Examples of questions	Probes
Informing on follow-up care	- According to you, what is your role in informing patients on follow-up care?<br>- According to you, what is your role in knowing what timely follow-up means for each abnormal result (lesion grade +- HPV result)<br>- What steps do you typically follow to ensure timely follow-up for these patients?<br>- What role do healthcare providers according to you play in encouraging patients to attend follow-up appointments?<br>- How do you ensure patients understand the urgency and importance of follow-up?	- Difference in role GP/gynaecologist?<br>- Do you use written materials or online resources to support your explanation?<br>- Do you modify your approach based on patient characteristics (e.g., health literacy level)?<br>- Do you coordinate with other professionals (GPs, gynaecologists, lab staff)?
Barriers to follow-up	- What challenges do you face in ensuring that patients receive timely follow-up care?<br>- What do you think contributes to delays in follow-up care, if you have observed any?<br>- According to you, what factors influence whether patients receive the recommended follow-up care timely?<br>- What concerns or misconceptions among patients regarding follow-up care have you encountered?	- Patient barriers: Financial constraints, employment status, education level<br>- Logistical barriers: staff shortage, time constraints, workload, administrative burden, waiting times<br>- Organizational barriers: size of practice, availability of specialists, geographical location
Recommendations for improving timely follow-up of abnormal cervical cancer screening results	- According to you, what resources or support systems do you believe would help addressing these barriers?<br>- From your perspective, how do you think untimely follow-up could be improved?	- Use of reminder systems, follow-up calls or any other strategies?

Healthcare system and policy-related influences on timely follow-up [Healthcare system and policy level]

*Objective: To identify healthcare providers' experiences with and perspectives on potential healthcare system and policy-related factors that might impact timely follow-up. **[individual: 10-15min; multiple: 15min]***

Topics	Examples of questions	Probes
Systemic and institutional barriers	- Which systemic and institutional factors, related to the cervical cancer screening programme, referral pathways, etc., do you think contribute to untimely follow-up?	- Systemic barriers: referral pathways, appointment availability, reimbursement policies, administrative workload<br>- Difference between urban and rural settings?
Awareness and effectiveness of the fail-safe mechanism	- What role do healthcare policies or institutional guidelines play in ensuring timely follow-up?<br>- Are you familiar with the fail-safe mechanism in place for the follow-up of cervical cancer screening? If yes, how effective do you think it is in ensuring timely follow-up?	- Have you seen cases where the fail-safe mechanism helped ensure follow-up?
Potential improvements to the system	- What improvements, if any, do you think could be made to the fail-safe mechanism or what could be added to the mechanism to reduce untimely follow-up?<br>- Which additional support mechanisms could enhance timely follow-up care, according to you?	

Closing the interview

*Objective: To thank the participant(s) and to finalize the conversation. **[individual: 5min; multiple: 10min]***

- Make sure all the topics are questioned and the information is complete
- Thank the participant(s) for their time and participation and let him/her/them know that you appreciate their contributions
- Provide time to exchange additional thoughts/questions after the interview
- Provide info on the next steps in the research project and follow-up
