# Supplementary material for: Untimely follow-up after cervical cancer screening: A qualitative study among healthcare providers in Flanders (Belgium)
Source: PLoS One. 2026 Oct 7;21(10):e0359860. doi: 10.1371/journal.pone.0359860 (PMC13645050; doi:10.1371/journal.pone.0359860)
Supplement: Supplementary file 2 [file pone.0359860.s002.pdf]

S2. Table. Coding frame with code definitions – interviews with healthcare providers.

Code	Definition code	Note/Consensus
**Patient-related factors**		Yellow = added at later stage
Socio-demographic and economic factors	Social and demographic factors that define the patient and that may contribute to delayed follow-up.	V-M: Add economic factors here as well, since low socioeconomic status is also included in this category.
Migration background	Migration background as a potential factor influencing the failure to follow up on abnormal screening results in a timely manner. Here, we are not referring so much to language (which is a separate factor), but primarily to cultural differences and the resulting lack of understanding or reluctance that may accompany them.	
Age	Older or younger patients who have different habits (such as preferring to see a gynecologist rather than a general practitioner, or vice versa) may be less likely to follow up on abnormal screening results in a timely manner.	
Language barrier	Not speaking the language or not understanding it well enough, which can lead to delays in follow-up.	
Relation- and partnerdynamics	Relationship and partner-related factors: partner history, fidelity/infidelity, stigma/guilt, emotions regarding sexual transmission.	
Health literacy	Encompasses more than just understanding and knowledge, which is why it is listed here as a main category: functional (reading, writing, and math skills), communication level (digital skills, reading comprehension, asking questions – and having the confidence to do so – of a healthcare provider, mobilizing	

	social support from friends and family), critical level (correctly interpreting health information, thinking ahead about the long-term consequences of health-related behavior, setting priorities, making decisions together with your healthcare provider).	
Knowledge of cervical cancer screening programme		K-M: This more specific subcode under health literacy is still missing here, as this is a common issue. It pertains to specific knowledge regarding cervical cancer screening and follow-up.
Knowledge of result and follow-up	Finding out the results of the Pap smear. Factors related to the patient herself that prevent her from being aware of the abnormal results (e.g., she doesn't answer the phone, or she isn't listening because she is overwhelmed by the information).	K-M: This code has been expanded to "Knowledge of results **and follow-up**": Knowing what the Pap smear result is and what the follow-up for this test entails. Factors related to the patient herself that prevent her from knowing or being aware of the abnormal result and/or follow-up (e.g., she does not answer the phone, or she does not listen because she is overwhelmed by the information.)<br><br>V-M: The definition of health literacy is actually reduced here to knowledge, which does not fully capture the essence of health literacy. Knowledge is one component of health literacy, so it can certainly be included here.<br>Knowledge regarding outcomes and/or follow-up does not mean simply being aware of the outcome or follow-up, but

		rather understanding the outcome. --> An important condition is that patients are informed about the result/follow-up but, for other reasons, do not grasp or understand this… if they are NOT informed, this belongs at a higher level (the healthcare provider has not informed them, they have not received the result via CoZo, etc.)
Knowledge digital healthcare platforms	The patient's familiarity with the various digital health platforms e.g., CoZo, MijnGezondheid, MyNexusHealth, VaccinNet… Whether or not the patient is familiar with these platforms and knows how and where information is displayed can be a factor influencing whether or not follow-up takes place in a timely manner.	
Behaviour and attitude	Behaviours and attitudes of the patient that can influence follow-up.	
Autonomy in screening and follow-up	The patient takes the initiative to manage their own follow-up care or, consciously or unconsciously, to facilitate the process (e.g. by providing the healthcare provider with the correct information), making independent decisions as a patient that may or may not influence that follow-up care.<br>--> Had a biopsy, followed by a few normal results, but annual follow-up remains advisable. The patient decides for themselves not to attend any further appointments/to stop attending	K-M: patient empowerment, e.g. the healthcare provider considers the patient capable of taking action themselves, asking for results, etc.<br><br>V-M: this definition is too narrowly formulated. We would also include, for example, whether or not the patient takes responsibility for updating their own details (telephone number, GP) where necessary.
Fear and misconceptions	Fear: Feelings of anxiety regarding results, follow-up examinations, and uncertainty.	

	Misconceptions: incorrect beliefs or ideas that are not accurate, regarding results, follow-up, or in general.	
Perception of time	For example “not realising that it was so long ago” + forgetfulness (losing track of time)	
Frequency of (GP) consultations	How often patients visit the doctor (the more often they visit, the easier it is to engage with them as a healthcare provider; women who visit more often tend to take greater control of their own health).	
(Personal) barriers	Personal factors relating to the patient that make timely follow-up difficult.	
Accessibility		K-M: Add a new subcode: ‘unaccessible. The patient does not answer the phone, or the telephone number/email address is incorrect, change of address, emails are not being read.<br><br>V-M: Use ‘accessibility as subcode instead of ‘unaccessible; do not specify the nature of the issue, so that both positive and negative situations can be included under this subcode.
Financial barrier	The cost of tests or healthcare providers’ fees as a factor that may prevent patients from receiving or seeking follow-up care, or from doing so in a timely manner.	
Unpleasant examination	For example, having to undergo that colposcopy time and time again and viewing it as an unpleasant examination; or viewing the Pap smear itself as an unpleasant examination, and as a result, not booking a follow-up appointment straight away.	
**Healthcare provider-related factors**		

Communication with patient	Overarching: Factors relating to communication between healthcare provider and patient.	
Communication about the result with the patient	Factors specifically relating to the communication of results between healthcare provider and patient.	
Communication method	The way in which the result is communicated: by letter, by email, by telephone, in person.	
Communication of abnormal result only	"No news = good news"	
Communication normal and abnormal result	When the healthcare providers communicates normal and abnormal results to the patient.	
Failure to report results (in a timely manner)	The healthcare provider does not provide the patient with the results (either because they have forgotten or for some other reason).	
Proactive communication	Proactive communication by the healthcare provider refers to all forms of proactive, anticipatory and patient-centred information exchange in which the healthcare provider takes the initiative to inform the patient or ask them about cervical screening, possible results and necessary follow-up.<br>This mainly covers three areas:<br>• Providing information in advance during a Pap smear<br>• Actively enquiring about screening status<br>• Actively enquiring about follow-up	
Address the complexity of the subject	Healthcare providers find it difficult to raise the subject, for various reasons (links to sexual activity, the complexity of managing various conditions in the associated follow-up care, etc.).	V-M: This refers not only to the socio-emotional aspects of an abnormal Pap smear result, but also to the technical aspects (the complexity of the type of abnormality).
Interdisciplinairy collaboration	Factors relating to collaboration between the various professionals involved in the follow-up of abnormal	

	screening results for cervical cancer (GPs, gynaecologists, nurses, midwives, administrative staff, etc.).	
Interdisciplinary communication	Factors specifically related to communication between actors.	
Communication of result between healthcare providers	Communication about the result of the Pap smear among healthcare providers involved in patient care.	
Communication of follow-up between healthcare providers	Communication about follow-up of the Pap smear among healthcare providers involved in patient care.	
Responsibility	Factors relating to the healthcare provider's responsibilities.	K-M: 'Responsibility' is used when the context explicitly refers to responsibility: "Who is considered responsible for what in the context of follow-up?"; when the context does not strictly refer to responsibility, we can use the code 'Task agreements'. There is some overlap here.
Smear taker is responsible for communicating with the patient	The healthcare provider who takes the Pap smear is responsible for communicating the result to the patient.	
Smear taker is responsible for follow-up	The healthcare provider who takes the Pap smear is responsible for the follow-up the abnormal result.	
Smear taker is other healthcare provider	The smear taker is a gynaecologist (if a GP) or a GP (if a gynaecologist), or the smear taker is a nurse.	
Patient responsibility	It is emphasised that the healthcare provider CONSIDERS follow-up to be the patient's responsibility.	

Task agreements	Factors relating to agreements between healthcare providers and all parties involved in the follow-up of abnormal screening results for CC, which either promote or hinder cooperation and communication.	K-M: ‘Responsibility’ is used when the context explicitly refers to responsibility: “Who is considered responsible for what in the context of follow-up?”; when the context does not strictly refer to responsibility, we can use the code ‘Task agreements’. There is some overlap here.
The healthcare provider arranges a follow-up appointment	The healthcare provider arranges the follow-up appointment for the patient, so that the patient does not have to make the appointment themselves.	
Continuity of care	How to best ensure continuity of care when changing healthcare providers, including preventive care.	V-M: when care needs to be taken over by another healthcare provider.
Organisation	Organisation at the individual level of the healthcare provider.	
Work pressure	(Too) much work as a factor that may influence the follow-up of abnormal results (not having or taking the time for this).	
Reminder to view results	The healthcare provider sets a reminder for themselves to check the results of the Pap smear x number of days later, as these always arrive with a delay because they first need to be analysed by the lab.	
Remider for follow-up	The healthcare provider sets a reminder for themselves to check whether the patient has attended their follow-up appointment, made an appointment, etc.	
Interpretation guidelines	The way in which the healthcare provider interprets the results (which may vary and lead to difficulties or problems in follow-up)	
Knowledge of fail-safe mechanism	Healthcare providers’ knowledge of the fail-safe mechanism.	
**Practice-related factors**		

Use of administrative systems	Barriers and recommendations relating to the medical programme fall under ‘Healthcare system and Policy’, whilst the effective use of administrative systems comes under ‘Practice-related factors’.	
Practice organisation	Organisation at the practice level (multiple healthcare providers, multidisciplinary approach, etc.).	K-M: Here we are talking specifically about coordination among colleagues within the practice.
Results followed up by a nurse or secretary	Factors relating to the handover of follow-up results to another member of staff (such as a nurse or secretary).	
Distribution of Pap smear taking in practice	The way in which the practice allocates the taking of Pap smears: does everyone do this, only/also a nurse, only female colleagues, etc.	Two codes combined into one: Smear tests taken by a nurse, Smear tests taken by a female colleague → this may also have an impact on whether follow-up is carried out in a timely manner.
**Healthcare system and policy-related factors**		
Guidelines for follow-up	Follow-up guidelines refer to the formal, policy-based recommendations and procedures that determine how abnormal results should be followed up.<br>These mainly include the following:<br>• Specific follow-up steps for each type of abnormal result<br>• Varying recommendations from different bodies (such as Sciensano, Domus Medica)<br>• Interdisciplinary (established) division of tasks and the lack of clear coordination (healthcare providers, laboratory, health insurance fund)	
New guidelines HPV	Specifically all matters relating to the new guidelines on primary HPV screening effective from January 1 2025	

Systemic barriers	Barriers within the healthcare system and policy that may affect timely follow-up.	
Legal factors	All legal, regulatory and professional ethical provisions that have an impact on the sharing, processing or follow-up of screening results. (GDPR, privacy, professional body rules)	
Screening invitation	Factors relating to the invitation to the screening programme for women when it has been too long since they had a smear test.	Relation to follow-up?
Type and timing of follow-up	• Type = nature of the further steps required following an abnormal result (colposcopy, repeat smear test)<br>• Timing = interval or waiting period that must be observed in accordance with guidelines or standard clinical practice	
Barriers to medical programmes	Any structural or functional limitations of the existing medical information system. (e.g. fragmentation of digital systems, lack of centralisation, technical limitations)	K-M: including digital health platforms such as CoZo
Waiting times specialist care	The amount of time it takes for a patient to secure an appointment with a gynaecologist can affect the timeliness of the follow-up appointment.	
Recommendations	Factors cited by healthcare professionals as recommendations for the healthcare system and policy that could improve timely follow-up.	K-M: If sending a physical letter to the woman's home is a common recommendation, we could create a specific subcode for this.
Division of tasks among actors	Policy agreements regarding the division of responsibilities among actors (e.g. prevention and screening in primary versus secondary care, fees, follow-up by the health insurance fund)	

Communication between healthcare providers		K-M: new code 'communication between healthcare providers' because we feel this is an important recommendation: communication between GPs and gynaecologists needs to be improved so that GPs are better informed about abnormal results via a report from the gynaecologist, rather than just seeing a lab result on CoZo.
Use of AI	Using AI to improve the follow-up of abnormal screening results.	
Recommendations medical programmes	Factors relating to the medical programme that the government could amend or add to improve the follow-up of abnormal results.	
Self-sample	Self-testing as a factor in improving follow-up care (not just screening).	
Patient health education	The government should educate patients (via social media, TV, leaflets/posters, etc.) to improve their health literacy and knowledge regarding cervical cancer screening, as well as the possible outcomes and follow-up procedures, so that this can lead to improved follow-up care.	
Comparing national screening programmes	When healthcare providers refer to, or compare with, other population-based screening programmes (breast cancer screening and colorectal cancer screening)	K-M: not just follow-up-related issues, but all matters relating to other population-based screening programmes and the comparison of cervical cancer screening.
Fail-safe mechanism	Factors relating to the fail-safe mechanism that are not linked to the healthcare provider's knowledge of the mechanism, but rather to the way the mechanism is	

	organised by the government (both positive and negative aspects).	
Information flows	All government information is disseminated to healthcare providers, patients and other stakeholders.	
Communication of result	Communication of the (abnormal) result to the healthcare provider or patient.	
Result via labo		K-M: This code relates to laboratory results, as well as the recommendations that are always provided alongside them. It also covers how healthcare professionals interpret these recommendations.
Communication result to patient	The results are communicated to the patient, not via the healthcare provider, but via the government.	K-M: The difference from the communication method (healthcare provider factors) is that this concerns the system (e.g. results sent to the patient via CoZo) rather than communication via the healthcare provider.
Communication result to healthcare provider	Factors relating to the way in which the results are communicated to the doctor, and how this flow of information is regulated by the government.	
Timing of receipt of results	E.g. The time between having a smear test and receiving the results; Two time points for result communication for cytology and HPV.	
Quantity of incoming information	Factors relating to the volume of information received by the healthcare provider. How this is organised at the level of the healthcare system.	
