# Supplementary material for: Untimely follow-up after cervical cancer screening: A qualitative study among healthcare providers in Flanders (Belgium)
Source: PLoS One. 2026 Oct 7;21(10):e0359860. doi: 10.1371/journal.pone.0359860 (PMC13645050; doi:10.1371/journal.pone.0359860)
Supplement: Supplementary file 5 [file pone.0359860.s005.docx]

Dear Editor,

Dear Reviewers,

We would like to thank you for the careful and detailed assessment of our revised manuscript. We are pleased that the majority of the revisions made in response to the first round of review have been well received, and we appreciate the constructive feedback provided in this second revision.

Below, we provide a point-by-point response to the remaining comments, along with a description of the changes made to the revised manuscript.

We hope that the revised manuscript and our point-by-point responses adequately address the remaining concerns, and we look forward to acceptance and publication of our manuscript.

Best wishes,

On behalf of all authors,

Marie Hendrickx

**Reviewer comments to the author:**

*Reviewer #1:  The manuscript is substantially improved and the Discussion and Conclusions are generally well aligned with the three stated study objectives. The factors contributing to untimely follow-up and healthcare providers’ recommendations for improving follow-up are clearly interpreted and appropriately contextualised. The first objective, concerning how the follow-up pathway unfolds from the healthcare providers’ perspective, is also addressed in the Discussion through the description of a fragmented, non-standardised, multi-step pathway with multiple handovers. However, this aspect could be slightly more explicitly developed as a finding in its own right.*

*A minor revision is therefore suggested in the Conclusion, where objectives 2 and 3 are clearly reflected, while objective 1 is less visible. Adding a brief statement summarising the characteristics of the follow-up pathway from the providers’ perspective would strengthen the correspondence between the study objectives, findings, and conclusions*

**We thank the reviewer for this comment. We agree that the first study objective, which is describing how the follow-up pathway unfolds from the healthcare providers' perspective, was less explicitly reflected in the conclusion compared to objectives 2 and 3. We have added a brief opening sentence to the conclusion summarising the characteristics of the follow-up pathway as experienced by healthcare providers: “*According to healthcare providers, the follow-up pathway after abnormal cervical cancer screening results is experienced as a complex, fragmented, and non-standardised multi-step process, characterised by multiple handovers, prolonged intervals, and heavy reliance on individual provider initiative rather than coordinated system-level structures.*” We believe this addition strengthens the correspondence between all three study objectives, the findings, and the conclusion, while maintaining the concise and focused nature of the concluding statement.**

*Reviewer #2: Dear Authors,*

*Thank you for the care you have taken in revising the manuscript. The response to the previous round of comments is thorough, and the additional methodological detail strengthens the transparency and credibility of the study considerably.*

*One point still needs to be addressed before the manuscript can be published:*

*• The proportion of women with inadequate follow-up is reported inconsistently across the manuscript. The Abstract states “approximately one in five women,” while the Introduction reports the specific figure of 17.7%. Please use a single, consistent figure and/or phrasing for this statistic throughout the manuscript (including the Abstract and any other section where it appears).*

*So, reconcile the abstract's "approximately one in five women" with the Introduction's 17.7% figure. Either use 17.7% consistently, or replace "one in five" with "approximately one in six" throughout, including in the new Data analysis reflexivity sentence.*

*This is a straightforward wording correction and does not require any new analysis. Once this is addressed, we anticipate the manuscript will be ready to move forward.*

*All the best!*

*Title: Untimely follow-up after cervical cancer screening: a qualitative study among healthcare providers in Flanders (Belgium)*

*Abstract*

*The abstract is partially addressed: The Abstract now reads "14 semi-structured group interviews... with 120 general practitioners and gynaecologists," removing the misleading N=120 framing. However, it now labels all 14 as "group" interviews, when 13 were group and 1 individual. Authors explain in their response that this detail was moved to the Methods for word-count reasons, a reasonable compromise, but the abstract sentence is still technically inexact.*

*"Approximately one in five women" (abstract) vs. 17.7% (Introduction) is an unreconciled inconsistency. This needs to be addressed before publication. The abstract is unchanged, and the phrase "one in five" has now also been added a second time.*

*, verbatim, in the new reflexivity paragraph in Data analysis — so the inconsistency has been reinforced rather than resolved. This is the one substantive fix still required.*

*Introduction*

*Same one-in-five vs. 17.7% inconsistency, viewed from the Introduction side. See Abstract above — 17.7% remains the only figure given in the Introduction; not cross-referenced to the abstract's "one in five."*

**We thank the reviewer for carefully identifying this inconsistency. We agree that the use of both “approximately one in five women” and “17.7%” across different sections of the manuscript was inconsistent and potentially confusing. We have replaced “approximately one in five women” with “17.7% of women” in both the abstract and the data analysis reflexivity paragraph, ensuring that a single, consistent figure is used throughout the manuscript. This brings these sections in line with the figure already reported in the introduction. We apologise for this oversight, which was introduced during the revision process.**

*Methods*

*-Group interviews (n=13) mislabelled as individual "semi-structured interviews" throughout. Fully corrected. Terminology updated throughout; a methodological/practical justification for the group format was added to Study design (p.6); the single individual interview's inclusion and joint analysis is explicitly justified (p.6–7); group-dynamic effects (social desirability, anchoring) are now addressed in Limitations.*

*-Missing researcher reflexivity/positionality statement (COREQ 8–9). A substantive reflexivity statement was added to both Data collection (interviewer's disciplinary background and prior CCS research experience, p.7–8) and Data analysis (prior knowledge, mitigation via double-coding and stakeholder revalidation, p.9). A dedicated trustworthiness paragraph (Lincoln & Guba criteria) was also added.*

*-Data saturation: A concrete, inductive criterion was added: no new codes generated across two consecutive group interviews (p.9).*

**We thank the reviewer for confirming that the corrections to group interview terminology, the addition of the reflexivity and positionality statement, the data saturation criterion, and the trustworthiness paragraph have been adequately addressed.**

*Results Ok*

*Table 1: GP subtotals summed to 92 against a stated n=91; grand total 119 ≠ 120. Clarified rather than corrected as an error: the nurse working alongside a GP is counted within the GP column, giving 92 (91 GPs + 1 nurse) + 28 gynaecologists = 120. The table header and accompanying sentence (p.8) now state this explicitly. Figures are internally consistent.*

*Recommendations section announces 5 themes but lists only 4. Corrected. The introductory sentence now consistently states four themes, matching the four subsections presented (p.15).*

**We thank the reviewer for confirming that Table 1 and the recommendations section have been resolved.**

*Discussion*

*Social desirability bias among providers self-reporting in peer groups was not acknowledged. Now explicitly named in the Discussion's opening paragraph and elaborated in a substantial new Limitations passage covering social desirability, self-serving attribution, and anchoring effects (p.19–20).*

*GP/gynaecologist sample imbalance should be framed as an explicit limitation, with mitigation noted. Well addressed: a new passage explains the imbalance and describes a concrete mitigation strategy (flexible, probing topic-guide application in gynaecologist interviews to ensure balanced theme coverage, p.19).*

*support the Netherlands/Denmark/UK vs. Germany/France/Belgium comparison with the 2025 Winterflood reference alongside or instead of the 2008 guidelines.*

*Implications ok*

**We thank the reviewer for confirming that the social desirability bias acknowledgement and the GP/gynaecologist sample imbalance have been well addressed. Regarding the suggestion to supplement or replace the 2008 European guidelines reference with the 2025 Winterflood reference for the specific comparison claim (lines 602–605), we have added the Winterflood reference of 2025 alongside the existing reference to better support this comparison with more recent evidence. We thank the reviewer for confirming that the implications section is satisfactory.**

*References*

*Some references were corrected.*

*-Reference 14 (Pangarkar, Bethesda System) incorrectly cited for a claim about Flemish follow-up rates. Corrected. The claim is now cited to the correct BCR/CvKO Jaarrapport 2022 report; the Pangarkar reference was removed from the list entirely, and all subsequent reference numbers were carefully and correctly renumbered throughout the manuscript.*

*-Two 2026-dated references cited despite data collection ending in November 2025. Authors clarify that these were incorporated during write-up (standard practice) and used only to contextualise interpretation, not to inform data collection/analysis. The PsyArXiv preprint reference now includes the full URL and access date per PLOS ONE policy.*

*-OSF Data Availability link pointed to the generic dashboard rather than the project page. Superseded by a more robust solution: the topic guide and coding frame are now provided as Supporting Information files (Table S1/S2) rather than via an OSF link, and a clear, specific pathway for transcript-access requests via the Ghent University Hospital ethics committee is provided.*

*However, replace/supplement the 2008 European guidelines reference with the 2025 Winterflood* *reference for the specific comparison claim. See Discussion above.*

**We thank the reviewer for confirming that the reference corrections, including the Pangarkar correction, the 2026-dated references, and the OSF data availability solution, have been adequately resolved. As mentioned above, we have added the Winterflood reference of 2025 alongside the existing reference to better support this comparison with more recent evidence.**

*Conclusion*

*The conclusion is clear, well-structured, and accurately reflects the findings without overstatement.*

**We thank the reviewer for the positive evaluation of the conclusion.**
