# Supplementary material for: Untimely follow-up after cervical cancer screening: A qualitative study among healthcare providers in Flanders (Belgium)
Source: PLoS One. 2026 Oct 7;21(10):e0359860. doi: 10.1371/journal.pone.0359860 (PMC13645050; doi:10.1371/journal.pone.0359860)
Supplement: Supplementary file 6 [file pone.0359860.s003.docx]

Dear Editor,

Dear Reviewers,

We would like to thank you for your thorough and constructive feedback, which has allowed us to substantially strengthen the methodological transparency and overall quality of the manuscript.

Below, we provide a point-by-point response to each comment, along with a description of the changes made to the revised manuscript.

We hope that the revised manuscript and our point-by-point responses adequately address the editor’s and reviewers' concerns, and we look forward to receiving your feedback.

Best wishes,
On behalf of all authors,

Marie Hendrickx

**Journal requirements:**

1. *Please ensure that your manuscript meets PLOS ONE's style requirements, including those for file naming. The PLOS ONE style templates can be found at*

*https://journals.plos.org/plosone/s/file?id=wjVg/PLOSOne_formatting_sample_main_body.pdf and*

[*https://journals.plos.org/plosone/s/file?id=ba62/PLOSOne_formatting_sample_title_authors_affiliations.pdf*](https://journals.plos.org/plosone/s/file?id=ba62/PLOSOne_formatting_sample_title_authors_affiliations.pdf)

**We thank the journal for this guidance. We have carefully reviewed the PLOS ONE style templates provided and have ensured that the revised manuscript adheres to all formatting requirements, including title and author formatting, heading styles, reference formatting, figure and table labelling, and file naming conventions. Any deviations from the required style identified during this review have been corrected in the revised submission.**

2. *Please include a complete copy of PLOS’ questionnaire on inclusivity in global research in your revised manuscript. Our policy for research in this area aims to improve transparency in the reporting of research performed outside of researchers’ own country or community. The policy applies to researchers who have travelled to a different country to conduct research, research with Indigenous populations or their lands, and research on cultural artefacts. The questionnaire can also be requested at the journal’s discretion for any other submissions, even if these conditions are not met.  Please find more information on the policy and a link to download a blank copy of the questionnaire here: https://journals.plos.org/plosone/s/best-practices-in-research-reporting. Please upload a completed version of your questionnaire as Supporting Information when you resubmit your manuscript.*

**We thank the journal for this requirement. We have completed the PLOS ONE inclusivity in global research questionnaire and uploaded it as Supporting Information with the revised submission. The fieldwork was conducted by researchers based in Belgium, within the same country and healthcare system as the study population. All fieldwork took place in Flanders, Belgium. We have completed the questionnaire as requested and are happy to provide any additional information if needed.**

3. *Thank you for stating the following financial disclosure:*

*“The study was conducted as part of the project Identification and prevention of social inequalities in the follow-up of abnormal cervical screening results, funded by the Research Foundation Flanders (FWO), within the framework of the ‘Kom op tegen Kanker’ campaign. Grant number: G0A7H24N”*

*Please state what role the funders took in the study. If the funders had no role, please state: "The funders had no role in study design, data collection and analysis, decision to publish, or preparation of the manuscript."*

*If this statement is not correct you must amend it as needed.*

*Please include this amended Role of Funder statement in your cover letter; we will change the online submission form on your behalf.*

**We thank the journal for this request. The funders had no role in study design, data collection and analysis, decision to publish, or preparation of the manuscript. We have included this statement below for incorporation in the cover letter and into the online submission form:**

***"The study was conducted as part of the project Identification and prevention of social inequalities in the follow-up of abnormal cervical screening results, funded by the Research Foundation Flanders (FWO), within the framework of the 'Kom op tegen Kanker (Stand up to Cancer)' campaign (Grant number: G0A7H24N). The funders had no role in study design, data collection and analysis, decision to publish, or preparation of the manuscript."***

4. *We note that this data set consists of interview transcripts. Can you please confirm that all participants gave consent for interview transcript to be published?*

**We thank the journal for this guidance** **and would like to clarify a possible misunderstanding. The dataset deposited at OSF does not contain interview transcripts, it comprises the coding frame with code definitions and the topic guide. No interview transcripts have been shared publicly, and none are included in the submission. Participants provided informed consent for their interview transcripts to be used for research purposes, however explicit consent for public sharing of the transcripts was not obtained in the informed consent form. As the transcripts contain detailed accounts of professional experiences and practices, even after pseudonymization there remains a risk that participants could be identifiable within their professional community, given the relatively small and specialised nature of the cervical cancer screening workforce in Flanders. For these reasons, and in accordance with the ethical approval granted by the UGent/Ghent University Hospital Medical Ethics Committee (reference number: ONZ-2025-0232), the interview transcripts cannot be made publicly available.**

**To support transparency and reproducibility, the full topic guide and the coding frame with code definitions have been included as Supporting Information with the revised submission. These materials provide sufficient detail to allow independent researchers to assess the analytical process and, where appropriate, replicate the study in other contexts.**

**The Data Availability statement has been updated to read as follows:**

***"The full topic guide and coding frame with code definitions are available as Supporting Information (Table S1 and S2). Interview transcripts cannot be made publicly available as explicit consent for public sharing was not obtained from participants, and sharing these data poses a risk to participant confidentiality given the specialised nature of the study population. Researchers who wish to request access to the transcripts may apply to the Ghent University Hospital / Ghent University Medical Ethics Committee, which assesses such requests independently of the authors (reference number: ONZ-2025-0232): Commissie voor Medische Ethiek, UZ Gent, C. Heymanslaan 10, 9000 Gent, Belgium; ethisch.comite@uzgent.be; tel. +32 9 332 33 36.”***

5. *Thank you for uploading your study's underlying data set. Unfortunately, the repository you have noted in your Data Availability statement does not qualify as an acceptable data repository according to PLOS's standards.*

*At this time, please upload the minimal data set necessary to replicate your study's findings to a stable, public repository (such as figshare or Dryad) and provide us with the relevant URLs, DOIs, or accession numbers that may be used to access these data. For a list of recommended repositories and additional information on PLOS standards for data deposition, please see* [*https://journals.plos.org/plosone/s/recommended-repositories*](https://journals.plos.org/plosone/s/recommended-repositories)*.*

**We thank the journal for this guidance. As the interview transcripts cannot be made publicly available, as explicit consent for public sharing was not obtained from participants and sharing poses a risk to participant confidentiality given the specialised nature of the study population, we are unable to upload a dataset to a public repository such as Figshare or Dryad.**

**To support transparency and reproducibility, all additional materials necessary to understand and assess the study, including the full topic guide and the coding frame with code definitions, have been included as Supporting Information with the revised submission. We believe these materials provide sufficient methodological detail to allow independent assessment of the analytical process and replication of the study in other contexts. The Data Availability statement has been updated accordingly.**

6. *We notice that your supplementary figures are uploaded with the file type 'Figure'. Please amend the file type to 'Supporting Information'. Please ensure that each Supporting Information file has a legend listed in the manuscript after the references list.*

**We thank the journal for this comment. However, we wish to clarify that the file uploaded in the initial submission was not a supplementary figure but rather the main manuscript figure (Fig 1), uploaded as a separate TIFF file in accordance with PLOS ONE's formatting requirements. No supplementary figures were included in the initial submission.**

**In the revised manuscript, we have uploaded two Supporting Information files (S1_File.pdf and S2_File.pdf), correctly labelled as 'Supporting Information' rather than 'Figure'. Legends for both Supporting Information files have been included in the manuscript after the references list, in accordance with PLOS ONE's requirements. The main manuscript figure (Fig 1) remains uploaded as a separate TIFF file as before.**

7. *If the reviewer comments include a recommendation to cite specific previously published works, please review and evaluate these publications to determine whether they are relevant and should be cited. There is no requirement to cite these works unless the editor has indicated otherwise.*

**We thank the journal for this clarification. We have carefully reviewed all publications recommended by the reviewers and have evaluated their relevance to the manuscript. Where deemed relevant and appropriate, this reference has been incorporated into the revised manuscript (Lincoln YS, Guba EG. Naturalistic Inquiry. Newbury Park, CA: Sage; 1985; framework to enhance trustworthiness in qualitative research). References that were considered not sufficiently relevant to the study's scope or findings have not been included.**

**Reviewer comments to the author:**

*Reviewer #1: This manuscript targets a critical vulnerability within cancer prevention frameworks by examining the operational and systemic friction points that delay follow-up diagnostic care after an abnormal cervical screening result. Given that nearly one in five women in Flanders experiences delayed or entirely absent follow-up care within the existing organized screening infrastructure, examining these clinical trajectories through the lens of frontline healthcare providers is highly relevant, as detailed in lines 95 to 98.*

*Methodologically, the study exhibits clear merits, as the use of an iterative thematic analysis with double coding strengthens the reliability of the findings, while the choice to collect and analyze data concurrently until empirical saturation was reached is entirely appropriate. By involving both general practitioners and gynaecologists within a multilevel framework, the analysis successfully demonstrates that structural failures, such as disconnected digital health records, ambiguous cross-disciplinary boundaries, and communication breakdowns, exert a far heavier toll on follow-up timelines than individual patient attributes.*

*Nevertheless, major contradictions regarding sample reporting and significant gaps in qualitative transparency must be resolved before the manuscript can align with the rigorous reporting standards of PLOS ONE. Primary among these concerns is an explicit numerical contradiction regarding the sample size between the abstract and the main text, where lines 29 and 30 of the abstract state that 14 semi-structured interviews were conducted in person with general practitioners and gynaecologists followed by an unexplained reference to an N of 120. This phrasing is deeply confusing because it remains fundamentally unclear whether the study is based on 14 individual interviews or an overall sample of 120 participants; if only 14 interviews occurred, the origin and meaning of the number 120 must be explicitly stated, whereas if 120 clinicians participated through other multi-person sessions, this directly undermines the description of individual semi-structured interviews.*

*The authors must reconcile these metrics and ensure absolute statistical consistency across the abstract, the methods section, and the demographic tables.*

**We thank the reviewer for flagging this inconsistency. The apparent contradiction arose from insufficiently clear reporting of the study design. The study comprised 14 semi-structured interviews in total, of which 13 were group interviews and one was an individual interview. In these 14 interviews, a total of 120 healthcare providers were included (resulting in an average of nine participants per group interview). This distinction was not made sufficiently explicit in the abstract, creating the confusion noted by the reviewer. We have revised the abstract to clarify that 14 group interviews were conducted with 120 general practitioners and gynaecologists in total. The minor detail that one interview was conducted individually rather than as a group interview has been added to the methods section rather than the abstract, as the abstract word limit of 250 words did not allow for this level of detail without exceeding the maximum or causing more confusion. We have ensured that the number of interviews, the group format, and the total number of participants are reported consistently and transparently throughout the manuscript.**

*Furthermore, the manuscript lacks an explicit declaration of its underlying ontological and epistemological assumptions, which is vital because qualitative data does not exist in a vacuum but is co-constructed based on the researchers' theoretical lens. The authors need to clearly state their paradigmatic positioning, such as constructivism, interpretivism, or critical realism, within the methods section to give readers a clear framework for how the themes were negotiated and interpreted.*

**We thank the reviewer for this valuable comment. We agree that making the underlying paradigmatic positioning explicit strengthens the transparency and rigor of the study. We have added a brief statement to the methods section declaring the study's interpretivist epistemological positioning, clarifying that participants' accounts were approached as subjective, contextually situated expressions of experience and meaning rather than objective representations of reality. This addition provides readers with a clearer framework for understanding how themes were developed and interpreted throughout the analytical process.**

**The study design section is updated to: “*This exploratory qualitative study is grounded in an interpretivist epistemological position, which holds that knowledge is inherently subjective and context-dependent. Rather than seeking a single objective account of the follow-up pathway, we aimed to understand how healthcare providers experience, interpret, and make meaning of this process within their specific professional and organisational contexts. To this end, semi-structured in-depth group interviews were conducted with healthcare providers responsible for cervical cancer screening in Flanders (Belgium) (i.e., GPs and gynaecologists). Group interviews were chosen as the primary data collection method for both methodological and practical reasons. Methodologically, the group format was considered particularly well-suited to exploring factors influencing follow-up, as peer discussion between clinicians can surface shared experiences, professional norms, and collegial dynamics that may not emerge as readily in individual interviews. Practically, interviews were organised through existing local quality assessment groups and professional associations, which are fixed, regularly meeting peer groups of healthcare providers, facilitating recruitment and enabling discussion within established professional relationships. One individual interview was additionally conducted with a GP working in a community health centre who expressed interest in participating but whose colleagues were not available to join. This interview was analysed together with the group data using the same analytical approach, as the topic guide and analytical framework were identical across all sessions. The data were analysed using a combined deductive-inductive thematic analysis approach. This approach allowed us to explore the pre-defined four-level perspective from the literature, while also capturing new insights from participants’ experiences. Accordingly, the themes generated through analysis reflect the researchers' interpretation of participants' perspectives, shaped by the analytical framework and the iterative dialogue between data and theory throughout the research process. This article was prepared in accordance with the consolidated criteria for reporting qualitative research (COREQ) (22). The study was approved by the UGent/Ghent University Hospital Medical Ethics Committee (reference number: ONZ-2025-0232).”***

*Alongside this philosophical framing, the level of researcher reflexivity remains superficial, and while the authors briefly acknowledge that data collection was conducted by a female, non-medical researcher trained in qualitative techniques, a rigorous qualitative study requires a critical evaluation of positionality.*

*The authors should expand on how the research team’s non-clinical identity, institutional backgrounds, and personal preconceptions may have shaped field rapport, influenced the responses of practicing medical specialists and general practitioners, or guided the final analytical coding.*

**We thank the reviewer for this important observation. We agree that a more explicit and critical reflection on researcher positionality strengthens the rigor and transparency of the study. We have expanded the reflexivity statement in the methods section (see data collection and data analysis) to address how the research team's backgrounds, institutional positioning, and prior knowledge may have shaped data collection and analysis, including both potential limitations and unexpected advantages.**

**Added to the data collection section, next to the main author being a public health researcher: *“The research team's non-clinical identity shaped the interview dynamic in both directions: participants may have withheld information or simplified clinical detail assuming it was beyond the interviewer’s grasp, but several also spontaneously elaborated on or repeated clinical concepts in greater depth than they might have with a clinician interviewer, potentially enriching the data.”***

**Added to the data analysis section: *“Reflexivity was considered throughout the analytical process. The primary researcher entered the study with familiarity with the broader screening context and knowledge that approximately one in five women do not receive timely follow-up, though the specific patient-, provider-, practice-, and system-level reasons underlying these delays were largely unknown prior to data collection. An iterative thematic analysis was conducted in MaxQDA, based on the six-step systematic process described by Naeem et al. (23). The multilevel framework (patient, provider, practice, healthcare system and policy) served as a deductive organisational structure, providing four pre-defined levels into which inductively generated codes were systematically assigned. Coding itself was conducted inductively, with themes and patterns emerging directly from the data and subsequently allocated to the most appropriate level of the framework. The inductive component in particular mitigated the influence of the researcher's preconceptions, as it allowed factors to emerge from the data itself rather than restricting the analysis to what was anticipated beforehand. Memos were used throughout the analytical process to document analytical thoughts, emerging interpretations, and code definitions, supporting transparency and consistency.***

***The coding frame was developed by two independent researchers (MH and LAK) based on approximately 10% of the data. The final coding frame, accompanied by code definitions, was then systematically applied to each interview. The coding frame with code definitions is available in Table S2. Each interview was coded by the primary coder (MH) and double-coded by a second researcher (KV, VV or LAK). Inter-coder agreement was established through an in-depth line-by-line, code-by-code discussion of each transcript between the two coders. Segments where the coders had applied different codes were then discussed in detail and discrepancies were resolved through discussion to reach consensus, ensuring that interpretations remained grounded in participants' own accounts rather than the researchers' prior assumptions. Finally, broader themes were generated from the codes and mapped onto the four categories of the framework. Data collection and analysis proceeded concurrently. Data saturation was assessed inductively by tracking the emergence of new codes across sessions. When no new codes emerged across two consecutive group interviews, it was determined that sufficient depth and breadth of information had been reached. All already planned interviews were conducted in full, but no additional interviews were scheduled once data saturation was reached. Findings were revalidated in a separate meeting with the stakeholder planning group, including policymakers and healthcare providers.”***

*Finally, although the authors note adherence to the COREQ guidelines, the actual operationalization of these standards needs a more transparent explanation, particularly where the text mentions that the interview guide was pilot-tested and received input from a stakeholder planning group. To ensure reproducibility, the authors must clarify who exactly composed this stakeholder group, how they were selected, and precisely how their feedback was integrated into either the analytical framework or the final validation of the data.*

**We thank the reviewer for this comment. We agree that greater transparency regarding the composition, selection, and role of the stakeholder planning group strengthens the reproducibility and rigour of the study. We have expanded the final paragraph of the data collection section of the methods to provide a more detailed account of who composed the group, how they were selected, and precisely how their input was integrated at each stage of the research process:**

***“Interviews were guided by a topic guide developed based on the study’s multilevel analytical framework, a review of the existing literature on follow-up of abnormal cervical cancer screening, and input from the stakeholder planning group. This group was recruited purposively through the professional network of the research team for the broader project and comprised representatives from BCR, CvKO, an academic and a clinical GP, a gynaecologist, and two women who had previously received an abnormal cervical cancer screening result. Group members reviewed a draft version of the topic guide during a dedicated meeting, with opportunity to suggest additional topics and adapt existing questions. The revised topic guide was subsequently pilot tested by one GP from the group through a simulated interview, allowing further refinement before formal data collection commenced.”***

*Reviewer #2: This is a fine paper on an interesting topic entitled: Untimely follow-up after cervical cancer screening: a qualitative study*

*The paper has the merit and has critical important findings among healthcare providers in Flanders. However, needs revision.*

*I will encourage the authors to download the COREQ checklist and use it to appraise their methods section in order to meet all the criteria for reporting a qualitative study.
All the best!*

*Title: Untimely follow-up after cervical cancer screening: a qualitative study among healthcare providers in Flanders (Belgium)*

*Abstract
Revise description of study design to correctly distinguish group interviews (n=13) from individual interviews (n=1) throughout the manuscript*

**We thank the reviewer for this comment. This issue was also raised by Reviewer #1 and has been addressed accordingly (p.5). We have revised the abstract to clarify that 14 group interviews were conducted with 120 general practitioners and gynaecologists in total. The minor detail that one interview was conducted individually rather than as a group interview has been added to the methods section rather than the abstract, as the abstract word limit of 250 words did not allow for this level of detail without exceeding the maximum or causing more confusion. We have ensured that the number of interviews, the group format, and the total number of participants are reported consistently and transparently throughout the manuscript.**

*Methods
Page 7, line 148: Misnaming of study design — group interviews treated as individual interviews. The manuscript refers throughout to ‘14 semi-structured interviews’. However, 13 of these were group interviews (average 9 participants), and only 1 was an individual interview. Methodologically, group interviews (Focus group) and individual (one-on-one) interviews are distinct methods that generate different types of data. This distinction is critical for the epistemological framing of the study. The manuscript should: (a) use the correct term (group interviews or focus group discussions) for the 13 group sessions; (b) briefly justify why group interviews were chosen as the primary method; (c) address in the limitations section how group dynamics may have influenced responses (e.g., social desirability within professional peer groups, anchoring effects); and (d) clarify whether the single individual interview was analysed differently or together with the group data.*

**We thank the reviewer for this important methodological observation. We agree that the distinction between group interviews and individual interviews is critical and should be made explicit throughout the manuscript. We have made the following changes:**

**(a) We have replaced "semi-structured interviews" with "semi-structured group interviews" throughout the manuscript where referring to the 13 group sessions, and clearly distinguished these from the single individual interview.**

**(b) We have added a brief justification for the choice of group interviews as the primary method to the study design section in Data & Methods (Study design): *“Group interviews were chosen as the primary data collection method for both methodological and practical reasons. Methodologically, the group format was considered particularly well-suited to exploring factors influencing follow-up, as peer discussion between clinicians can surface shared experiences, professional norms, and collegial dynamics that may not emerge as readily in individual interviews. Practically, interviews were organised through existing local quality assessment groups and professional associations, which are fixed, regularly meeting peer groups of healthcare providers, facilitating recruitment and enabling discussion within established professional relationships. One individual interview was additionally conducted with a GP working in a community health centre who expressed interest in participating but whose colleagues were not available to join. This interview was analysed together with the group data using the same analytical approach, as the topic guide and analytical framework were identical across all sessions. The themes identified in this interview were consistent with those raised in the group sessions, and no codes were unique to it, supporting its inclusion in the combined analysis.”***

**(c) We have added a reflection on how group dynamics may have influenced responses to the limitations section: *“Although the group interview format was chosen deliberately to stimulate peer discussion and capture shared professional perspectives, it may also have introduced group dynamic effects. Social desirability within professional peer groups may have led some participants to align their responses with dominant views within their group, avoid disclosing practices deviating from professional norms, or attribute delays to external system- and policy-level factors rather than to their own clinical practice. Anchoring effects, whereby early contributions from more vocal or senior participants shape the direction of subsequent responses, cannot be excluded. While the presence of a second researcher taking field notes allowed for some observation of non-verbal communication and group dynamics, these potential influences should also be considered when interpreting the relative emphasis placed on different levels of the multilevel framework.”***

**(d) We have clarified in the methods section that the individual interview was analysed together with the group data using the same analytical approach: “*One individual interview was additionally conducted with a GP working in a community health centre who expressed interest in participating but whose colleagues were not available to join. This interview was analysed together with the group data using the same analytical approach, as the topic guide and analytical framework were identical across all sessions.”***

*Absence of a researcher reflexivity and positionality statement. COREQ item 8 requires a statement on the relationship between the interviewer and participants, and COREQ item 9 requires disclosure of interviewer characteristics (professional background, qualifications, experience).*

*Page 8, line 161: The manuscript notes the lead interviewer was ‘non-medical, trained in qualitative interview techniques’ but does not address broader reflexivity: how the researchers’ disciplinary backgrounds may have shaped data collection, question framing, or interpretation. A dedicated reflexivity paragraph in the Methods section is needed.*

**We thank the reviewer for these valuable comments. The absence of a reflexivity and positionality statement was also raised by Reviewer #1, and has been addressed accordingly (p.6-7). We have added a reflexivity statement to both the data collection and data analysis sections of the manuscript. In the data collection section, the description of the primary researcher has been expanded beyond her qualification as a non-medical researcher trained in qualitative techniques to include her disciplinary background as a public health researcher with prior experience in cervical cancer screening research. We additionally reflect on how the research team's non-clinical identity may have influenced the interview dynamic, noting that while this may have shaped rapport with practicing healthcare providers, it also proved advantageous in practice, as several participants elaborated on clinical concepts in greater depth than they might have with a clinician interviewer. In the data analysis section, we acknowledge that the primary researcher entered the study with limited prior knowledge of the screening context, including awareness that approximately one in five women do not receive timely follow-up, while the specific patient-, provider-, practice-, and system-level reasons underlying these delays were largely unknown. We address how the potential influence of these preconceptions on question framing and interpretation was mitigated through a combined deductive-inductive analytical approach, double-coding by two independent researchers, iterative discussion to resolve discrepancies, and final revalidation of findings with the stakeholder planning group. These additions directly address COREQ items 6, 7 and 8. Please refer to our response to Reviewer #1 for full details of the changes made.**

*Insufficient explanation on data saturation.*

*The manuscript states that ‘data collection and analysis proceeded concurrently until data saturation was reached’ but does not describe how saturation was assessed. With 13 group sessions and 120 participants, saturation is not self-evident, especially given that group interactions can surface diverse views rapidly. Did the researchers apply a formal saturation criterion (e.g., no new codes after a defined number of sessions)? Was reflexive saturation or theoretical saturation used? Clarifying this strengthens the methodological rigour claim.*

**We thank the reviewer for this important observation. We agree that a clearer description of how saturation was assessed strengthens the methodological rigor of the study. We have revised the relevant sentence in the data analysis section of Data & Methods to describe the saturation approach and the practical criterion used to determine when saturation had been reached. Specifically, data saturation was assessed inductively by tracking the generation of new codes across sessions. When no new codes were generated across two consecutive group interviews, it was determined that sufficient depth and breadth of information had been reached. All planned interviews were still conducted, but no additional interviews were scheduled beyond those:**

***“Data saturation was assessed inductively by tracking the generation of new codes across sessions. When no new codes were generated across two consecutive group interviews, it was determined that sufficient depth and breadth of information had been reached. All already planned interviews were conducted in full, but no additional interviews were scheduled once data saturation was reached.”***

*Results
Critical data error: Page 7, line157: Table 1 participant counts are internally inconsistent.
The stated sample is N = 120 (GPs n = 91, gynaecologists n = 28; 91 + 28 = 119, not 120). Furthermore, all within-category subtotals for GPs consistently sum to 92, not 91:
• Gender: Male 37 + Female 55 = 92 (stated: 91)
• Years of experience: 4 + 16 + 30 + 19 + 23 = 92 (stated: 91)
• Practice location: Rural 62 + Urban 30 = 92 (stated: 91)
• Practice type: 19 + 61 + 10 + 2 = 92 (stated: 91)
This systematic discrepancy of 1 in every GP subtotal, combined with the overall total mismatch (119 vs. 120), strongly suggests a data entry error in the table. The authors must verify and correct all participant counts and reconcile with the N = 120 stated in the abstract and methods. If the nurse participant is creating ambiguity in categorisation, this must be explicitly clarified.*

**We thank the reviewer for the careful scrutiny of Table 1. We want to clarify that there is no data error. The apparent inconsistency arises from insufficient clarity in the reporting of the sample composition, which we have now improved.**

**One nurse working in a GP practice participated in the study alongside a participating GP, as was mentioned in the data collection section. This nurse was counted together with the GPs in Table 1, resulting in a column total of 92 (91 GPs + 1 nurse). This explains why all GP subcategory subtotals consistently sum to 92 rather than 91. The overall total of 120 is therefore correct and consistent throughout the manuscript: 92 (GPs + 1 nurse) + 28 gynaecologists = 120.**

**The nurse's participation was mentioned in the data collection section of the manuscript, but we acknowledge that this was not stated clearly enough to prevent confusion when reading Table 1. We have therefore made the following changes to improve clarity:**

- **The column header in Table 1 has been adjusted to: "General practitioners and nurse (n=92), of whom 91 GPs and 1 nurse in a GP practice"**
- **The sentence in the data collection section has been revised to more explicitly state that the nurse is included in the GP category in Table 1:**

***"Among these, one nurse who assisted a participating GP, two retired healthcare providers, and six healthcare providers in training participated, as they were still actively involved in the practice, networks or associations by contributing to cervical cancer screening activities. Participant characteristics are shown in Table 1, the nurse is included in the GP category, resulting in a combined total of 92 for that column."***

*Page 18, lines 427-431: The fourth theme in the recommendations section is described as optimising the fail-safe mechanism, and this is listed as item '4)' in the introductory sentence. However, earlier in that same sentence, five themes are announced: '1) proactive and standardised communication... 2) clear task allocation... 3) integrated and transparent digital systems... and 4) optimising the fail-safe mechanism.' Only four themes are listed in the sentence, yet five are mentioned in the accompanying text. Please reconcile and ensure the number of themes stated matches the content presented.*

**We thank the reviewer for identifying this inconsistency. This error arose during an earlier revision of the manuscript, in which the recommendations section was restructured from five to four themes by integrating one theme into the remaining ones. The introductory sentence was not fully updated to reflect this change, resulting in a mismatch between the stated number of themes (five) and the themes actually presented (four). We have corrected the introductory sentence to consistently state four themes throughout, ensuring full alignment between the announced structure and the content presented.**

*Discussion
The discussion is thorough and appropriately contextualises findings within international literature. The acknowledgement that provider perspectives may not fully capture patient-level barriers is commendable and epistemically honest. The proposal to shift primary smears to primary care is well-grounded in literature and avoids overstatement. The transition to HPV screening is appropriately integrated as a contextual factor.*

*However:
Social desirability bias is not acknowledged. The study relies entirely on healthcare providers’ self-reported perceptions of why follow-up is delayed. Providers described patient-level factors as secondary compared to system-level and provider-level ones. This framing may reflect genuine system dysfunction, but it could equally reflect social desirability bias (providers may be reluctant to acknowledge their own role in delays within a peer group setting, particularly in group interviews where professional reputation may be at stake).
The discussion acknowledges only the absence of patient perspectives but does not address the potential for self-serving attribution (i.e., attributing delays to external factors rather than individual practice). This limitation should be explicitly named in the limitations’ subsection.*

**We thank the reviewer for this important observation. We agree that the potential for social desirability bias and self-serving attribution in provider self-reports is a meaningful limitation that should be explicitly acknowledged. While the discussion already notes that the absence of patient perspectives limits the completeness of the picture, it does not sufficiently address the possibility that providers may have been inclined, consciously or unconsciously, to attribute delays to external system- and policy-level factors rather than to their own clinical practice, particularly within a group interview setting where professional reputation may be at stake. We have added an explicit acknowledgement of this limitation to the limitations section (also based on the comment on group interviews in Data & Methods).**

**Added in limitations section: *“Although the group interview format was chosen deliberately to stimulate peer discussion and capture shared professional perspectives, it may also have introduced group dynamic effects. Social desirability within professional peer groups may have led some participants to align their responses with dominant views, avoid disclosing practices deviating from professional norms, or attribute delays to external system- and policy-level factors rather than to their own clinical practice. Anchoring effects, whereby early contributions from more vocal or senior participants shape the direction of subsequent responses, cannot be excluded. While the presence of a second researcher taking field notes allowed for some observation of non-verbal communication and group dynamics, these potential influences should be considered when interpreting the relative emphasis placed on different levels of the multilevel framework.”***

*Implications
The following implications are clearly supported by the data and well-articulated:
• Standardising communication protocols at the time of smear collection, including prepared patient-facing explanations of possible results and follow-up steps.
• Clarifying responsibility for follow-up between GPs and gynaecologists, ideally through formal agreements or system-embedded task allocation.
• Investing in integrated digital infrastructure: centralised screening history modules within electronic medical records, automated reminders at evidence-based intervals, and uniform result transmission standards (e.g., HL7-FHIR).
• Optimising and expanding the fail-safe mechanism to include patient-directed notifications alongside provider alerts.

Increasing primary care engagement in initial cervical cancer screening to reduce overreliance on specialist capacity and to leverage GPs’ longitudinal patient relationships.
References Page 26, line 679: Reference 14 appears incorrectly matched to its citation. The manuscript states (p. 5): 'In-depth analyses from the Belgian Cancer Registry (BCR) and the CvKO demonstrate that follow-up rates in Flanders vary according to the cytological and associated HPV result (14).' Reference 14 is: Pangarkar MA. 'The Bethesda System for reporting cervical cytology.' Cytojournal. 2022;19:28.
This paper describes a classification system for cytological reporting — it does not contain data on follow-up rates in Flanders. The correct citation for this claim should be a Belgian Cancer Registry or CvKO report (e.g., ref. 7 or ref. 13). Please verify and correct.*

**We thank the reviewer for the positive evaluation of the implications section and for confirming that these are clearly supported by the data and well-articulated. In addition, we thank the reviewer for carefully identifying this reference error. We have corrected the reference error as described above, and reference 14 (Pangarkar et al., 2022) has been replaced by reference “*Centre for Cancer Detection, Belgian Cancer Registry, Department of Care. Jaarrapport 2022. 2022*” for the claim regarding follow-up rates in Flanders varying according to cytological and associated HPV result. Pangarkar et al., 2022 is not further used in the paper and therefore excluded from the reference list. All other citations have been carefully reviewed to ensure correctness and consistency throughout the manuscript.**

*References dated 2026 (refs. 25 and 33) were cited in a manuscript reporting data collection completed November 2025. Ref. 25 (Balamou et al., Prev Med Rep. 2026;62:103396) and ref. 33 (ten Hoor et al., PsyArXiv, 2026) both post-date or coincide with the stated data collection end date. For ref. 25, please clarify if this article was available in press or as an early online publication during the analysis period. For ref. 33, which is a PsyArXiv preprint, PLOS ONE's policy on citing preprints should be confirmed; if allowed, the preprint URL and access date must be included.*

**We thank the reviewer for this comment. We wish to clarify that while data collection was completed in November 2025, the analysis, interpretation, and write-up of the manuscript continued beyond this date, as is standard practice in qualitative research. References 26 (Balamou et al. 2026) and 34 (Ten Hoor were identified and incorporated during the write-up phase, which extended into 2026. Their inclusion in the discussion is therefore appropriate, as they were used to contextualise and support the interpretation of findings rather than to inform data collection or analysis. There is no inconsistency between the data collection end date and the publication dates of references cited in the discussion.**

**Regarding reference 33 (ten Hoor et al., PsyArXiv, 2026), we have verified that PLOS ONE permits the citation of preprints and have updated the reference to include the full preprint URL and access date in accordance with PLOS ONE's guidelines: *“Ten Hoor GA, Peters GJ, Crutzen R, Fernández ME, Markham C, Ruiter RAC, et al. Nuancing “Knowledge” as a Target for Behavior Change Interventions: Implications for Effective Intervention Design. PsyArXiv. 2026. Available from: https://osf.io/preprints/psyarxiv/6gbpa_v1 [Accessed 11 May 2026].”***

*Conclusion
The conclusion is clear, well-structured, and accurately reflects the findings without overstatement.
The 'double safety net' framing is effective and provides a memorable conceptual contribution.
The final sentence appropriately flags the ongoing relevance of findings to the HPV-primary screening context.*

**We thank the reviewer for this positive evaluation of the conclusion. We are pleased that the 'double safety net' framing was found to be effective and that the conclusion was considered to accurately reflect the findings without overstatement. Therefore, no changes were made to the conclusion in response to this comment.**

*Reviewer #3:*

*Overall, this is a well-conducted and relevant qualitative study that addresses an important gap in the literature on timely follow-up after abnormal cervical cancer screening. The manuscript is generally clearly written, uses a sound multilevel framework, and provides useful insights from healthcare providers’ perspectives. The large sample size and stakeholder validation are notable strengths. However, there are several areas where the manuscript could be strengthened, particularly in methodological transparency, reporting rigor, and balance in the discussion of limitations. Below, I outline my specific comments section by section.*

*Study population and recruitment*

*A notable limitation is the sampling approach. Although this is a qualitative study, the authors primarily relied on convenience sampling through professional networks and emails, with only a brief mention of subsequent purposive sampling to include providers from community health centres. In qualitative research, purposive sampling (with clear selection criteria) is the standard to ensure information-rich participants and sufficient variation for in-depth understanding (Patton, 2015; Creswell & Poth, 2018).*

*The description lacks detail on the specific purposive criteria used (e.g., years of experience, practice setting, geographic diversity, or experience with vulnerable populations), how maximum variation was achieved, and how saturation was monitored. Heavy reliance on convenience sampling risks selection bias and may limit the richness and transferability of the findings.
Recommendation: Strengthen the description of the purposive sampling strategy, explicit inclusion criteria, and efforts to achieve diversity in the Methods section. Also, acknowledge this limitation more clearly in the Discussion/Limitations.*

**We thank the reviewer for this important methodological observation. We agree that a clearer and more detailed description of the sampling strategy strengthens the transparency and rigor of the study. We wish to clarify and expand on the sampling approach as follows:**

**Initial recruitment was conducted through broad outreach across Flanders, targeting all GP associations and local quality assessment groups in Flanders, as well as the Flemish Organisation for Obstetrics and Gynaecology (VVOG), ensuring that all GPs and gynaecologists professionally active in Flanders were reached. This broad, region-wide outreach ensured a wide geographic spread of participants across all provinces of Flanders. Participation was based on willingness and availability, which introduced an element of convenience; however, the inclusive nature of the outreach mitigated selection bias by maximising the pool of potential participants. Furthermore, as interviews were conducted within existing local quality assessment groups (i.e., fixed peer groups of healthcare providers), natural variation in participant characteristics was achieved within each group, including variation in gender, years of experience, frequency of performing cervical cancer screening, and practice setting (solo practice, group practice, hospital-based). Purposive sampling was subsequently applied when it became apparent that GPs working in community health centres, who serve a disproportionately high share of socially disadvantaged and vulnerable populations, were underrepresented in the sample. Five GPs working in a community health centre were therefore specifically recruited to ensure that perspectives relevant to this patient population were captured:**

***“Eligible participants were GPs and gynaecologists actively involved in cervical cancer screening in Flanders. Recruitment was conducted through broad outreach targeting all GP associations across Flanders, and local quality assessment groups of GPs and gynaecologists, as well as the Flemish Organisation for Obstetrics and Gynaecology, ensuring geographic diversity across all provinces and natural variation in participant characteristics (gender, years of experience, practice setting). Participation was based on willingness and availability, introducing an element of convenience sampling. Purposive sampling was subsequently used to ensure inclusion of healthcare providers working in community health centres, as these settings are known to serve a disproportionately high share of vulnerable and socially disadvantaged populations and were not included yet in the sample.”***

**We acknowledge that the predominance of convenience-based recruitment is a limitation, and have added an explicit acknowledgement of this to the limitations section: *“Although broad outreach across all GP associations and gynaecological networks in Flanders ensured wide geographic coverage and natural variation in participant characteristics, recruitment was primarily based on willingness and availability, which introduces an element of convenience sampling. Providers who were more motivated or engaged with the topic of cervical cancer screening may have been more likely to participate, potentially introducing self-selection bias. This should be considered when assessing the transferability of the findings.”***

*Data Collection*

*The authors conducted 14 semi-structured interviews (13 group interviews and 1 individual), involving a total of 120 healthcare providers, with an average of 9 participants per group interview. While this large sample size strengthens the breadth and transferability of the findings and is commendable for a qualitative study, it also raises some concerns. In qualitative research, particularly with group interviews, very large group sizes can sometimes limit the depth of individual contributions and reduce opportunities for rich, nuanced exploration of experiences.
The authors have provided a reasonable justification for the sample size (achieving broad geographical and professional representation and continuing until saturation). However, the manuscript would benefit from additional discussion regarding the group dynamics in the interviews. For example, how did the relatively large group size influence participation patterns, power dynamics, or the emergence of dominant voices? How did the facilitators manage these dynamics to ensure all participants (including those in training or with less experience) could contribute meaningfully?*

*Recommendation: Expand the Data Collection section to briefly address how group size and composition affected data quality and interaction. Also, consider adding a short reflection on this issue in the Limitations section*

**We thank the reviewer for this comment. The influence of group dynamics on data quality was also raised by Reviewer #2 and has been addressed accordingly (p.13). We have added a description of how group size and composition may have influenced participation patterns and data quality to the data collection section: *“Group interviews were chosen as the primary data collection method for both methodological and practical reasons. Methodologically, the group format was considered particularly well-suited to exploring factors influencing follow-up, as peer discussion between clinicians can surface shared experiences, professional norms, and collegial dynamics that may not emerge as readily in individual interviews. Practically, interviews were organised through existing local quality assessment groups and professional associations, which are fixed, regularly meeting peer groups of healthcare providers, facilitating recruitment and enabling discussion within established professional relationships. One individual interview was additionally conducted with a GP working in a community health centre who expressed interest in participating but whose colleagues were not available to join. This interview was analysed together with the group data using the same analytical approach, as the topic guide and analytical framework were identical across all sessions. The themes identified in this interview were consistent with those raised in the group sessions, and no codes were unique to it, supporting its inclusion in the combined analysis.”***

**Furthermore, we have explicitly acknowledged the potential for social desirability bias, anchoring effects, and dominant voices in the limitations section: *“Although the group interview format was chosen deliberately to stimulate peer discussion and capture shared professional perspectives, it may also have introduced group dynamic effects. Social desirability within professional peer groups may have led some participants to align their responses with dominant views, avoid disclosing practices deviating from professional norms, or attribute delays to external system- and policy-level factors rather than to their own clinical practice. Anchoring effects, whereby early contributions from more vocal or senior participants shape the direction of subsequent responses, cannot be excluded. While the presence of a second researcher taking field notes allowed for some observation of non-verbal communication and group dynamics, these potential influences should also be considered when interpreting the relative emphasis placed on different levels of the multilevel framework.”***

*Data Analysis*

*The data analysis section is generally well-described and demonstrates good methodological rigor through double-coding, iterative analysis, and stakeholder validation. However, several aspects could be strengthened:*

*• The integration of deductive (multilevel framework) and inductive approaches is mentioned but not sufficiently detailed. It would be helpful to clarify how initial deductive codes were applied, how inductive codes were generated and merged, and the decision-making process for finalizing the coding frame.*

*• While double-coding is a strength, information on inter-coder agreement (even if qualitative) or the number of rounds needed to reach consensus would enhance transparency.
• The achievement of data saturation is stated but lacks operational detail (e.g., whether thematic, code, or meaning saturation was used, and in which specific interviews no new insights emerged).
• The use of MaxQDA is noted, but there is limited description of how specific features of the software supported the analysis (e.g., queries, memos, or visualization tools).*

*Additionally, a notable omission in this qualitative study is the absence of a dedicated section or subsection addressing rigor/trustworthiness (credibility, transferability, dependability, and confirmability). In qualitative research, it is standard practice to explicitly discuss strategies used to enhance trustworthiness (e.g., member checking, audit trail, reflexivity, thick description) following Lincoln and Guba (1985) or similar frameworks. The authors have incorporated some of these elements (double-coding, stakeholder validation, OSF availability), but they are scattered throughout the text rather than systematically presented. Adding a concise “Trustworthiness of the Findings” or “Rigor” subsection would significantly improve methodological transparency and align with best practices in qualitative reporting (e.g., COREQ guidelines).*

*Recommendation: Expand the Data Analysis section with more procedural detail and include a dedicated paragraph or subsection on rigor/trustworthiness.*

**We thank the reviewer for these detailed and constructive observations. We have addressed each sub-point as follows:**

- **Integration of deductive and inductive approaches: We have clarified in the data analysis section that the multilevel framework (patient, provider, practice, healthcare system and policy) served as a deductive organisational structure, providing four predefined levels into which inductively generated codes were systematically assigned. Coding itself was conducted inductively, with codes emerging from the data and subsequently allocated to the most appropriate level of the framework. This clarification better reflects the actual analytical process: *“The multilevel framework (patient, provider, practice, healthcare system and policy) served as a deductive organisational structure, providing four pre-defined levels into which inductively generated codes were systematically assigned. Coding itself was conducted inductively, with themes and patterns emerging directly from the data and subsequently allocated to the most appropriate level of the framework. The inductive component in particular mitigated the influence of the researcher's preconceptions, as it allowed factors to emerge from the data itself rather than restricting the analysis to what was anticipated beforehand. Memos were used throughout the analytical process to document analytical thoughts, emerging interpretations, and code definitions, supporting transparency and consistency.”***
- **Inter-coder agreement: We have added a clarification that coding by the two coders was compared line-by-line and code-by-code across each full transcript. Segments where the coders had applied different codes were then discussed in detail until consensus was reached, with each coder substantiating their interpretation with reference to the transcript. This process typically took two to three hours per interview, and all discrepancies were resolved within this single round, so no further coding rounds were required: *“Inter-coder agreement was established through an in-depth line-by-line, code-by-code discussion of each transcript between the two coders. Segments where the coders had applied different codes were then discussed in detail and discrepancies were resolved through discussion to reach consensus, ensuring that interpretations remained grounded in participants' own accounts rather than the researchers' prior assumptions.”***
- **MaxQDA features: We have added a brief description of how specific MaxQDA features supported the analysis, including the use of memos to document analytical thoughts and code definitions: *“An iterative thematic analysis was conducted in MaxQDA, based on the six-step systematic process described by Naeem et al. (23). The multilevel framework (patient, provider, practice, healthcare system and policy) served as a deductive organisational structure, providing four pre-defined levels into which inductively generated codes were systematically assigned. Coding itself was conducted inductively, with themes and patterns emerging directly from the data and subsequently allocated to the most appropriate level of the framework. The inductive component in particular mitigated the influence of the researcher's preconceptions, as it allowed factors to emerge from the data itself rather than restricting the analysis to what was anticipated beforehand. Memos were used throughout the analytical process to document analytical thoughts, emerging interpretations, and code definitions, supporting transparency and consistency.”***
- **Data saturation: This was addressed in our response to Reviewer #2. Data saturation was assessed inductively by tracking the generation of new codes across sessions. When no new codes were generated across two consecutive group interviews, saturation was considered reached: *“Data saturation was assessed inductively by tracking the generation of new codes across sessions. When no new codes were generated across two consecutive group interviews, it was determined that sufficient depth and breadth of information had been reached. All already planned interviews were conducted in full, but no additional interviews were scheduled once data saturation was reached.”***

**We thank the reviewer for the comment on the trustworthiness subsection. We agree that the trustworthiness strategies, while present throughout the manuscript, were not systematically presented. We have added a dedicated trustworthiness paragraph at the end of the data analysis section, explicitly addressing credibility, transferability, dependability, and confirmability following Lincoln and Guba's (1985) framework: *“To enhance the trustworthiness of the findings, several strategies were applied following Lincoln and Guba (24) framework (25). Credibility was supported through iterative concurrent data collection and analysis, double-coding of all transcripts with in-depth line-by-line consensus discussions, stakeholder revalidation of findings, and active researcher reflexivity throughout the process, in which the primary researcher’s prior knowledge of the screening context and non-clinical background were made explicit and revisited. Transferability was addressed through thick description of the study context and sample, and broad recruitment across all provinces of Flanders and across primary and secondary care providers. Dependability was strengthened through a systematic six-step thematic analysis process with a transparently developed coding frame and explicit code definitions, all available in Table S2. Finally, confirmability was supported through the use of memos to document analytical decisions throughout the process, and stakeholder validation to verify that findings were grounded in participants' accounts rather than researcher assumptions.”***

*The Limitations section is generally well-written and demonstrates appropriate reflexivity. The authors correctly acknowledge the lack of patient perspectives, the over-representation of GPs, and the context-specific nature of the findings. However, some statements could be strengthened. For example, the justification for having more GPs than gynaecologists is reasonable, but the potential bias this introduces (e.g., stronger emphasis on primary care barriers) could be discussed more critically. Additionally, while the large sample size is presented as a strength, in a qualitative study it may also have compromised depth in some group interviews — this trade-off could be briefly addressed.*

*Recommendation: Refine the Limitations to provide a more critical reflection on how the sampling imbalance and large group sizes may have influenced the findings, while maintaining the positive tone regarding diversity.*

**We thank the reviewer for these observations. The concerns raised regarding the influence of large interview group sizes on participation patterns, depth of individual contributions, and group dynamics, including social desirability bias, anchoring effects, and dominant voices, has been explicitly addressed in the limitations section in response to Reviewer #1 and #2 (p.5-6, 9).**

**Regarding the comment on sampling imbalance between GPs and gynaecologists, we acknowledge this limitation and agree that it warranted more critical discussion. We have elaborated the existing paragraph on sample composition in the limitations section, explicitly acknowledging that the overrepresentation of GPs may have introduced a greater emphasis on primary care perspectives and barriers, and that findings relating to secondary care should be interpreted with this in mind: *“Although most cervical smears are performed by gynaecologists in Flanders (7), the sample included more GPs than gynaecologists, partly due to challenges in recruiting specialists. To mitigate this risk of disproportionately shaping the findings, the topic guide was applied flexibly during interviews with gynaecologists, with the facilitator actively probing topics and perspectives that had emerged prominently in GP interviews but had not yet been spontaneously raised, ensuring that all key themes were meaningfully explored across both provider groups.”***
