# Supplementary material for: Untimely follow-up after cervical cancer screening: A qualitative study among healthcare providers in Flanders (Belgium)
Source: PLoS One. 2026 Oct 7;21(10):e0359860. doi: 10.1371/journal.pone.0359860 (PMC13645050; doi:10.1371/journal.pone.0359860)
Supplement: Supplementary file 7 [file pone.0359860.s004.docx]

**Comments**

| **Comments to the editor** | Dear Editors,  To the Editors,  Regarding the revised manuscript "Untimely follow-up after cervical cancer screening: a qualitative study among healthcare providers in Flanders (Belgium)" (PONE-D-26-26213):  I recommend **Accept** pending *Minor Revision* before publication.  The authors have engaged substantively and thoroughly with the prior round of comments, and the manuscript is methodologically sound and makes a valuable contribution to an under-studied area in the European context.  One outstanding issue remains, however: the manuscript reports the proportion of women with inadequate follow-up inconsistently. The Abstract states "approximately one in five women," while the Introduction gives the specific figure of 17.7%, both referring to the same underlying statistic.  This is a wording inconsistency rather than a substantive concern, but it should be reconciled to a single, consistent figure/phrasing throughout the manuscript before publication.  This does not require new analysis or another round of external peer review. Once the authors submit a corrected version harmonizing this figure, the manuscript should be ready for editorial acceptance and publication. |
| --- | --- |
| **Comments to the authors** | Dear Authors,  Thank you for the care you have taken in revising the manuscript. The response to the previous round of comments is thorough, and the additional methodological detail strengthens the transparency and credibility of the study considerably.  One point still needs to be addressed before the manuscript can be accepted:  • The proportion of women with inadequate follow-up is reported inconsistently across the manuscript. The Abstract states “approximately one in five women,” while the Introduction reports the specific figure of 17.7%. Please use a single, consistent figure and/or phrasing for this statistic throughout the manuscript (including the Abstract and any other section where it appears).  So, reconcile the abstract's "approximately one in five women" with the Introduction's 17.7% figure. Either use 17.7% consistently, or replace "one in five" with "approximately one in six" throughout, including in the new Data analysis reflexivity sentence.  This is a straightforward wording correction and does not require any new analysis. Once this is addressed, we anticipate the manuscript will be ready to move forward.  All the best! |
| **Manuscript Title** | Untimely follow-up after cervical cancer screening: a qualitative study among healthcare providers in Flanders (Belgium) |
| **Abstract** | The abstract is partially addressed: **The Abstract now reads "14 semi-structured group interviews... with 120 general practitioners and gynaecologists," removing the misleading N=120 framing**. However, it now labels all 14 as "group" interviews, when 13 were group and 1 individual. Authors explain in their response that this detail was moved to the Methods for word-count reasons, a reasonable compromise, but the abstract sentence is still technically inexact.  "Approximately one in five women" (abstract) vs. 17.7% (Introduction) is an unreconciled inconsistency. This needs to be addressed before publication. The abstract is unchanged, and the phrase "one in five" has now also been added a second time.  , verbatim, in the new reflexivity paragraph in Data analysis — so the inconsistency has been reinforced rather than resolved. This is the one substantive fix still required. |
| **Introduction** | Same one-in-five vs. 17.7% inconsistency, viewed from the Introduction side. See Abstract above — 17.7% remains the only figure given in the Introduction; not cross-referenced to the abstract's "one in five." |
| **Methods** | -Group interviews (n=13) mislabelled as individual "semi-structured interviews" throughout. Fully corrected. Terminology updated throughout; a methodological/practical justification for the group format was added to Study design (p.6); the single individual interview's inclusion and joint analysis is explicitly justified (p.6–7); group-dynamic effects (social desirability, anchoring) are now addressed in Limitations.  -Missing researcher reflexivity/positionality statement (COREQ 8–9). A substantive reflexivity statement was added to both Data collection (interviewer's disciplinary background and prior CCS research experience, p.7–8) and Data analysis (prior knowledge, mitigation via double-coding and stakeholder revalidation, p.9). A dedicated trustworthiness paragraph (Lincoln & Guba criteria) was also added.  -Data saturation: A concrete, inductive criterion was added: no new codes generated across two consecutive group interviews (p.9). |
| **Results** | Ok  Table 1: GP subtotals summed to 92 against a stated n=91; grand total 119 ≠ 120. Clarified rather than corrected as an error: the nurse working alongside a GP is counted within the GP column, giving 92 (91 GPs + 1 nurse) + 28 gynaecologists = 120. The table header and accompanying sentence (p.8) now state this explicitly. Figures are internally consistent.  Recommendations section announces 5 themes but lists only 4. Corrected. The introductory sentence now consistently states four themes, matching the four subsections presented (p.15). |
| **Discussion** | Social desirability bias among providers self-reporting in peer groups was not acknowledged. Now explicitly named in the Discussion's opening paragraph and elaborated in a substantial new Limitations passage covering social desirability, self-serving attribution, and anchoring effects (p.19–20).  GP/gynaecologist sample imbalance should be framed as an explicit limitation, with mitigation noted. Well addressed: a new passage explains the imbalance and describes a concrete mitigation strategy (flexible, probing topic-guide application in gynaecologist interviews to ensure balanced theme coverage, p.19).  support the Netherlands/Denmark/UK vs. Germany/France/Belgium comparison with the 2025 Winterflood reference alongside or instead of the 2008 guidelines. |
| **Implications** | ok |
| **References** | Some references were corrected.  -Reference 14 (Pangarkar, Bethesda System) incorrectly cited for a claim about Flemish follow-up rates. Corrected. The claim is now cited to the correct BCR/CvKO Jaarrapport 2022 report; the Pangarkar reference was removed from the list entirely, and all subsequent reference numbers were carefully and correctly renumbered throughout the manuscript.  -Two 2026-dated references cited despite data collection ending in November 2025. Authors clarify that these were incorporated during write-up (standard practice) and used only to contextualise interpretation, not to inform data collection/analysis. The PsyArXiv preprint reference now includes the full URL and access date per PLOS ONE policy.  -OSF Data Availability link pointed to the generic dashboard rather than the project page. Superseded by a more robust solution: the topic guide and coding frame are now provided as Supporting Information files (Table S1/S2) rather than via an OSF link, and a clear, specific pathway for transcript-access requests via the Ghent University Hospital ethics committee is provided.  However, replace/supplement the 2008 European guidelines reference with the 2025 Winterflood reference for the specific comparison claim. See Discussion above — not fully addressed. |
| **Conclusion** | The conclusion is clear, well-structured, and accurately reflects the findings without overstatement. |

Comments to the authors

Dear Authors,

Thank you for the care you have taken in revising the manuscript. The response to the previous round of comments is thorough, and the additional methodological detail strengthens the transparency and credibility of the study considerably.

One point still needs to be addressed before the manuscript can be accepted:

• The proportion of women with inadequate follow-up is reported inconsistently across the manuscript. The Abstract states “approximately one in five women,” while the Introduction reports the specific figure of 17.7%. Please use a single, consistent figure and/or phrasing for this statistic throughout the manuscript (including the Abstract and any other section where it appears).

So, reconcile the abstract's "approximately one in five women" with the Introduction's 17.7% figure. Either use 17.7% consistently, or replace "one in five" with "approximately one in six" throughout, including in the new Data analysis reflexivity sentence.

This is a straightforward wording correction and does not require any new analysis. Once this is addressed, we anticipate the manuscript will be ready to move forward.

All the best!

**Manuscript Title**: Untimely follow-up after cervical cancer screening: a qualitative study among healthcare providers in Flanders (Belgium)

**Abstract**

The abstract is partially addressed: The Abstract now reads "14 semi-structured group interviews... with 120 general practitioners and gynaecologists," removing the misleading N=120 framing. However, it now labels all 14 as "group" interviews, when 13 were group and 1 individual. Authors explain in their response that this detail was moved to the Methods for word-count reasons, a reasonable compromise, but the abstract sentence is still technically inexact.

"Approximately one in five women" (abstract) vs. 17.7% (Introduction) is an unreconciled inconsistency. This needs to be addressed before publication. The abstract is unchanged, and the phrase "one in five" has now also been added a second time.

, verbatim, in the new reflexivity paragraph in Data analysis — so the inconsistency has been reinforced rather than resolved. This is the one substantive fix still required.

Introduction	Same one-in-five vs. 17.7% inconsistency, viewed from the Introduction side. See Abstract above — 17.7% remains the only figure given in the Introduction; not cross-referenced to the abstract's "one in five."

**Methods**

-Group interviews (n=13) mislabelled as individual "semi-structured interviews" throughout. Fully corrected. Terminology updated throughout; a methodological/practical justification for the group format was added to Study design (p.6); the single individual interview's inclusion and joint analysis is explicitly justified (p.6–7); group-dynamic effects (social desirability, anchoring) are now addressed in Limitations.

-Missing researcher reflexivity/positionality statement (COREQ 8–9). A substantive reflexivity statement was added to both Data collection (interviewer's disciplinary background and prior CCS research experience, p.7–8) and Data analysis (prior knowledge, mitigation via double-coding and stakeholder revalidation, p.9). A dedicated trustworthiness paragraph (Lincoln & Guba criteria) was also added.

-Data saturation: A concrete, inductive criterion was added: no new codes generated across two consecutive group interviews (p.9).

**Results**	Ok

Table 1: GP subtotals summed to 92 against a stated n=91; grand total 119 ≠ 120. Clarified rather than corrected as an error: the nurse working alongside a GP is counted within the GP column, giving 92 (91 GPs + 1 nurse) + 28 gynaecologists = 120. The table header and accompanying sentence (p.8) now state this explicitly. Figures are internally consistent.

Recommendations section announces 5 themes but lists only 4. Corrected. The introductory sentence now consistently states four themes, matching the four subsections presented (p.15).

**Discussion**

Social desirability bias among providers self-reporting in peer groups was not acknowledged. Now explicitly named in the Discussion's opening paragraph and elaborated in a substantial new Limitations passage covering social desirability, self-serving attribution, and anchoring effects (p.19–20).

GP/gynaecologist sample imbalance should be framed as an explicit limitation, with mitigation noted. Well addressed: a new passage explains the imbalance and describes a concrete mitigation strategy (flexible, probing topic-guide application in gynaecologist interviews to ensure balanced theme coverage, p.19).

support the Netherlands/Denmark/UK vs. Germany/France/Belgium comparison with the 2025 Winterflood reference alongside or instead of the 2008 guidelines.

**Implications**	ok

**References**

Some references were corrected.

-Reference 14 (Pangarkar, Bethesda System) incorrectly cited for a claim about Flemish follow-up rates. Corrected. The claim is now cited to the correct BCR/CvKO Jaarrapport 2022 report; the Pangarkar reference was removed from the list entirely, and all subsequent reference numbers were carefully and correctly renumbered throughout the manuscript.

-Two 2026-dated references cited despite data collection ending in November 2025. Authors clarify that these were incorporated during write-up (standard practice) and used only to contextualise interpretation, not to inform data collection/analysis. The PsyArXiv preprint reference now includes the full URL and access date per PLOS ONE policy.

-OSF Data Availability link pointed to the generic dashboard rather than the project page. Superseded by a more robust solution: the topic guide and coding frame are now provided as Supporting Information files (Table S1/S2) rather than via an OSF link, and a clear, specific pathway for transcript-access requests via the Ghent University Hospital ethics committee is provided.

However, replace/supplement the 2008 European guidelines reference with the 2025 Winterflood reference for the specific comparison claim. See Discussion above — not fully addressed.

**Conclusion**

The conclusion is clear, well-structured, and accurately reflects the findings without overstatement.
